# European beech spring phenological phase prediction with UAV-derived multispectral indices and machine learning regression

**DOI:** 10.1038/s41598-024-66338-w

**Published:** 2024-07-09

**Authors:** Stuart Krause, Tanja Sanders

**Affiliations:** 1 Thünen Institute of Forest Ecosystems, Alfred-Möller-Str. 1, Haus 41/42, 16225 Eberswalde, Germany; 2 Department of Geography, University of Bonn, Meckenheimer Allee 166, 53115 Bonn, Germany

**Keywords:** Phenology, UAV, Machine learning, Intensive forest monitoring, Environmental sciences, Optics and photonics

## Abstract

Acquiring phenological event data is crucial for studying the impacts of climate change on forest dynamics and assessing the risks associated with the early onset of young leaves. Large-scale mapping of forest phenological timing using Earth observation (EO) data could enhance our understanding of these processes through an added spatial component. However, translating traditional ground-based phenological observations into reliable ground truthing for training and validating EO mapping applications remains challenging. This study explored the feasibility of predicting high-resolution phenological phase data for European beech (*Fagus sylvatica*) using unoccupied aerial vehicle (UAV)-based multispectral indices and machine learning. Employing a comprehensive feature selection process, we identified the most effective sensors, vegetation indices, training data partitions, and machine learning models for phenological phase prediction. The model that performed best and generalized well across various sites utilized Green Chromatic Coordinate (GCC) and Generalized Additive Model (GAM) boosting. The GCC training data, derived from the radiometrically calibrated visual bands of a multispectral sensor, were predicted using uncalibrated RGB sensor data. The final GCC/GAM boosting model demonstrated capability in predicting phenological phases on unseen datasets within a root mean squared error threshold of 0.5. This research highlights the potential interoperability among common UAV-mounted sensors, particularly the utility of readily available, low-cost RGB sensors. However, considerable limitations were observed with indices that implement the near-infrared band due to oversaturation. Future work will focus on adapting models to better align with the ICP Forests phenological flushing stages.

## Introduction

First proposed by the Swedish botanist in his work *Philosphia Botinica* in 1751^1^, the concept of gathering data on the timing of leaf opening, flowering, fruiting, and leaf fall alongside climatological observations “so as to show how areas differ” is still relevant today^2^. Historically phenological observations assisted in agriculture by means of predicting the timing of cultivation practices^3^ and emerged as a scientific discipline in the last 100 years^4^. Recently recognized as bioindicators of climate change^5^, phenological data proofs a sensitive proxy for climate change investigation^4^ due to the observed relationship between phenological timing and changing climate. In particular, spring phenology mirrors the changing temperatures^6^. Understanding phenological variations at the stand level provides insights into early spring flushing advances and the risk of late frost damage. This historical foundation sets the stage for contemporary research, which is now increasingly focused on the impacts of recent climatic shifts as highlighted by the Intergovernmental Panel on Climate Change (IPCC).

The IPCC reported a 1.53°C increase in average land temperature for the period 2006–2015 in comparison to the 1850–1900 period (IPCC, 2018). Warmer temperatures, alongside changing precipitation patterns, altered the growing seasons, causing increased tree mortality (IPCC, 2018); however, warmer temperatures may also lead to increased carbon storage due to longer growing seasons^7^. These extended by approximately 10–20 days in recent decades, and projected temperature increases of 1.4–5.8 °C in the next century may benefit some species while threatening others^8^. On of these threatened species is European beech (*Fagus sylvatica* L.), which is now experiencing shifts in phenological events with significant ecological consequences.

In central Europe European beech faces a potential threat from earlier budbreak and leaf-unfolding with extended growing seasons. Sudden freezing temperatures, known as late frost, can damage newly unfolded leaves and affect growth^9–11^. Late frost damage shortens the growing season and occurs predominantly in younger beech stands without an upper canopy layer according to reports by Sachsenforst (Pirna, Germany). This highlights the need for phenological modeling at a regional scale to identify high-risk areas for adaptive forest management practices. Such ecological impacts underscore the need for advanced phenological modeling that can predict and mitigate potential adverse outcomes, especially in regions prone to extreme climatic events.

Temporal phenological models typically rely on the timing of phenological events recorded as the day of the year (DOY)^12^. Phenological models also consider temperature-dependent chilling days and thermal time, which can be species-specific^13^. However, accurate models should account for other factors, such as photoperiod and precipitation^14^. Accurate phenological models are essential as early warning systems for stressed forest ecosystems and for simulating phenological processes across various timeframes. They are vital for grasping the spatial and temporal differences in forest phenology at a regional scale, offering insights into the extent of climate change and variations in carbon fluxes^15^. Accurate modeling requires robust datasets, which are primarily derived from detailed phenological observations at the ground level, despite certain limitations.

Visual phenological observations conducted by experts through long-term ground observation schemes provide valuable data at the stand and individual tree levels^6,16,17^. While subjective, these observations are considered highly accurate and provide information on crown conditions and potential tree damage. However, visual ground observations are labor intensive, limited in spatial coverage, and require experienced observers. Standardized observations at intensive monitoring plots, such as those conducted by ICP Forests, therefore provide valuable information on individual tree conditions^16^.

Terrestrial observation methods using webcams and automated cameras mounted on towers have become popular for monitoring forest phenology. These methods provide quantitative multispectral data that accurately capture the timing of phenological events. However, their spatial coverage is limited, and they are often used to validate satellite observation platforms^18^ and assist in mapping and modeling phenological metrics globally^19^. While ground observations are invaluable, technological advancements expanded our capabilities, enabling the use of satellites and UAVs to complement and enhance terrestrial data collection.

Satellite-based remote sensing has also become a valuable tool for studying phenology at different spatial scales^20^. Open access satellite platforms such as Landsat 8 and Sentinel 2a/b provide global coverage with temporal and spatial resolutions of 16 days and 30 m (Landsat, 2022) and 5 days and 10 m (Copernicus, 2022), respectively. The MODIS Global Land Cover Dynamics Product offers land surface phenology information on a global scale at a 500 m spatial resolution^21,22^. However, linking plot-level measurements to satellite-derived pixel values is challenging due to phenological heterogeneity and other factors^23,24^. Unoccupied Aerial Vehicles (UAVs), in particular, bridge the spatial resolution gap between extensive satellite surveillance and detailed terrestrial observations, offering a nuanced view of phenological dynamics.

The utilization of UAVs, has increasingly become a key tool for enhancing phenological observations in recent years, bridging the gap between terrestrial and satellite-based phenological observation systems. One major challenge is that a typical terrestrial observation plot often covers only a limited number of satellite pixels. Berra^25^ highlighted that within a single Landsat pixel, phenological events can show significant variance (R^2^ < 0.50) when compared to UAV-derived phenometrics. Moreover, satellite pixels may not adequately account for microclimatic variability^23^, which is especially relevant in areas where tree phenology is not uniform. This suggests that UAV data, when trained from localized observation plots, could be scaled up to encompass larger areas, thus enhancing the training scope for more satellite pixels. Along these lines, Atkins et al.^26^ demonstrated that UAVs and terrestrial camera systems could be effectively used in conjunction to gather high-resolution phenological data. The data collected via UAVs not only provide high-resolution insights but also present unique challenges and opportunities for applying modern automated techniques such as machine learning.

Converting UAV data into phenological metrics presents challenges, including sensor calibration and processing workloads for analysis-ready datasets. Flight campaigns aim to determine the onset of spring leafing out and acquire training data representative of the complete phenophase range. The number of flight missions depends on the required phenophase resolution and may involve repeated missions at various observation plots^27^. Automatic methods and explainable ML algorithms are crucial for organizing and processing the acquired data. However, expert-based qualitative methods are necessary for assessing the complexities of phenological development.

ML algorithms, particularly for image classification, gained popularity in remote sensing applications. They including support vector machines, random forests, and neural networks and offer higher accuracy than traditional parametric methods^28^. ML techniques incorporating features such as vegetation indices and meteorological data can spatially analyse the influence of temperature and precipitation on phenological processes^29^. UAV-based ML modeling has been applied to monitor individual tropical tree phenology using various RGB-based textures and vegetation indices^30^. Building on these technological and methodological advancements, our study employs UAV-derived multispectral data and ML to refine the extraction and analysis of phenological phases in European beech.

In this study, we investigate the potential of using UAV-based multispectral data and ML algorithms to automate the extraction of phenological phases for European beech. Initially, we examined phenological trends from 2006 at the Britz research station to provide an overview of specific patterns. Subsequently, we analyse multispectral data and derived indices from 2019 to 2021, conducting correlation analysis and polynomial fitting with field observations for feature selection. Additionally, ML techniques in regression mode are employed to train models using data from 2019 and 2020, which are then tested against the unobserved spring phenological phases of 2021. Ultimately, the chosen model is trained with various data subsets categorized by year of origin and thoroughly tested on new data from 2022, as well as data from older beech stands (over 50 years) and a different region. Unique to this study is the rigorous feature selection process, designed to replicate and preserve decades of expert knowledge of phenological ground observations into generalizable, region-based predictive models.

## Methods

### Study site

UAV and ground-based phenological observations for this study were carried out at the Britz intensive forest monitoring research station (https://deims.org/8ee82a9b-5086-4547-b5aa-4064e3314762) located in the lowlands of northeastern Brandenburg, Germany. Brandenburg lies between oceanic and continental climate zones and belongs to the young moraine landscape of the *Weichsel* glacial period. The soil at the site is locally known as a “*Finowtaler Sandbraunerde*”^31^ and is rated as a *Dystric Cambisol* derived from *Pleistocene* sand deposits with a *moderate* organic layer^32^. The average yearly temperature and precipitation for the region have been recorded at approximately 8.9 °C and 570 mm^33^, respectively. Datasets acquired from other sites, for the purpose of testing models with unseen data, were acquired from “Kahlenberg” near the Britz research station and the “Black Forest” located north of Freiburg in southwestern Germany.

Initially designed in 1972 for intensive forest hydrological research the Britz research station features nine large-scale lysimeters planted with a mixture of deciduous and coniferous species (Müller 2010). The site is equipped with an array of digital and analog dendrometers and various other sensors for sapflow and soil moisture measurements as well as meteorological data. Recently, the research station underwent a major digitalization overhaul with sensor data automatically being synced to a cloud and individual trees mapped at subdecimeter accuracy.

### Phenological ground observations

Ground-based phenological observations started in 2006 on all nine plots as well as at various satellite plots using traditional methods. UAV-based phenological missions started at the research site since 2018 with the aid of on-board multispectral sensors. As of 2021, phenological observations are additionally carried out with tower-based phenology cameras. Prior to 2020, traditional observation methods and UAV-based missions were conducted with a minimum offset of ± 3 days between them. Starting in 2020, traditional observations and UAV missions were carried out synchronously, with no offset.

**Figure 1:**
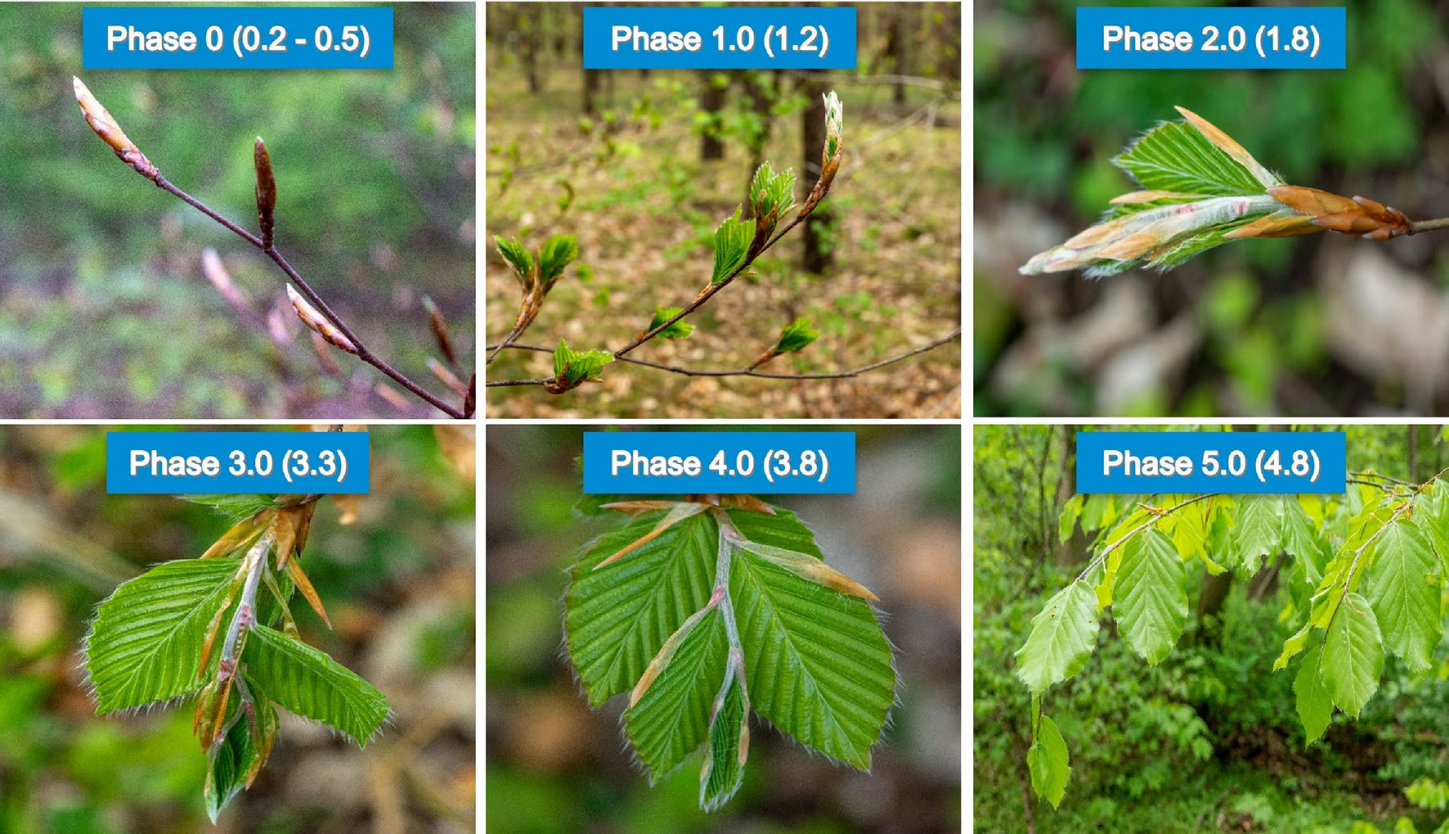
Sequential stages of spring phenology in a European beech (Fagus sylvatica) stand. Phase 0 (0.2–0.5) depicts bud swelling. Phase 1.0 (1.2) shows bud burst. Phase 2.0 (1.8) illustrates young leaves still enclosed by stipules. Phase 3.0 (3.3) captures the expansion of leaves, with stipules starting to fall off. Phase 4.0 (3.8) represents young fully expanded leaves, and Phase 5.0 (4.8) shows mature leaves. All images were captured in a single stand located in Britz, Germany, representing the typical phenological progression in early spring over a period of several weeks.

The traditional terrestrial phenological observations for beech focuses on the spring green-up phases (0–5) and fall senescence (discoloration and foliation). The spring phases consist of five phases based on various observation techniques, emphasizing early bud development and leaf hardening. The phases range from 0.0 to 5.0, with decimal values used to indicate progress between phases. For example, if 80% of the observed tree crown is in phase 4, it is recorded as phase 3.8. The *Britzer* method of spring phenological phase assessment is unique in the sense that it emphasizes the early onset of bud development with reference to the swelling of buds (phase 0.5) in early spring as well as the hardening and darkening of leaves in phase 5. Table 1 describes the various phases implemented with the *Britzer* method alongside other well-known techniques. The differentiation between phase 4.0 and 5.0 is implemented with the *Britzer* method and only considered with the Malisse/Schüler steps^34,35^. Figure 1 gives a photographic representation of the *Britzer* method phases.

**Table 1:** Overview of the various tree phenology observation methods for spring leafing out.

Forstreuter (stages)	Malaisse/Schüler (steps)	LFE (phases)	ICP forests (Flushing)	Britzer (phases)	Description
0A	1	1	1	0	Buds established from previous year
0A	1	1	1	0.2	Buds in Winter dormancy
0B	2	2	1	0.5	Swelling
0C	2	2	2	0.8	First buds are bursting (ICP Forests “flushing” = infrequent or slight)
1D	3	3	2–4	1.0	Budburst (full tree)
2E	4	4	2–4	(1.5)	
3	5	5	5	2.0	Young wrinkled leaf visible
–	6	–	–	3.0	Young leaf less wrinkled and long shoot begins to lengthen
–	6	–	–	4.0	Long shoot fully developed and pilose. Leaves still soft and pilose
–	7	–	–	5.0	Long shoot almost glabrous and leaves hardened as well as dark green, less pilose

Alongside the phenological phases, the *Britzer* method also takes the foliation percentage into account (0–100%), which is not directly harmonizable with the ICP Forests flushing method^16^. The ICP Forests method is shown in Table 1 for comparison purposes only and should not be taken as a direct method for conversion. Importantly, the *Britzer* method records foliation as the coverage in % of fully developed leaves, whereas the ICP Forests method of flushing records the coverage of green foliation and cannot be directly harmonized.

### UAV multispectral image acquisition

The UAV remote sensing platform used for this study comprised two Unoccupied Aerial Systems (UAS) customized for dual sensor RGB and multispectral image acquisition. The UAS implemented in 2019 comprised a *DJI Phantom 4 Professional Obsidian* (dji.com) with an RGB sensor (mechanical shutter) and a *Micasense Rededge-MX* multispectral sensor mounted with a custom 3D-printed gimbal (droneparts.de). The UAS implemented after 2020 was a *DJI Martice 210 RTK* (dji.com) with built-in Real-Time-Kinematic capabilities that applies real-time corrections for enhanced positional accuracy. Mounted on the *Matrice 210 RTK* was a *Zenmuse X7* RGB sensor alongside a *Micasense Altum* (micasense.com) multispectral sensor. Specific sensor details for the two multispectral and two RGB sensors can be found in Table 2. Table 3 displays the wavelength and bandwidth information for both multispectral sensors. The longwave infrared (LWIR) band was not implemented for this study.

**Table 2:** Overview of the sensor parameters used in this study.

Sensor	Mega-pixel	Focal length [mm]	Pixel size [μm]	Sensor size [mm]	Sensor size [pixel]	Aspect	File type	GSD [cm] at 75 m
DJI phantom 4 Pro (hasselblad)	20	24	3.09	13.2 × 8.8	5472 × 3648	3:2	jpeg	2.64
Micasense rededge-MX	1.2	5.4	3.75	4.8 × 3.6	1280 × 960	4:3	tif	5.27
DJI zenmuse X7	24	24	3.91	23.5 × 15.7	6016 × 4008	3:2	jpeg	1.23
Micasense altum	3.2 0.02	8 (multi) 1.77 (LWIR)	4.25 11.9	7.16 × 5.35 1.9 × 1.43	2064 × 1544 160 × 120	4:3 4:3	tif	4.33 52.48

**Table 3:** Wavelength and bandwidth for the Micasense Rededge-MX and Altum multispectral sensors.

Band name	Micasense altum		Micasense rededge-MX	
	Center wavelength [nm]	Bandwidth [nm]	Center wavelength [nm]	Bandwidth [nm]
Blue	475	32	475	20
Green	560	27	560	20
Red	668	14	668	10
Red edge	717	12	717	10
Near IR	842	57	840	40
LWIR	11 μm	6 μm	–	–

The phenology-based image acquisition starts shortly before budburst when buds begin swelling (0.5) and thereafter cycling in a maximum of 3 days during the fast-developing phases of 0.5 to 3. After the third phase, flights and ground observations are limited to biweekly acquisition days. Flight missions were carried out near solar noon (± 90 min), and calibration panel images were taken before and after missions as well as the acquisition of downwelling light sensor (DLS) information for each individual multispectral image. After 2020, special care was taken to acquire calibration panel images during moments when the sun was the least affected by cloud cover, especially when the sun was revealed during the flight mission. To ensure the capture of as many phases as possible, flight missions were carried out regardless of cloud cover, refrained from during precipitation and, for the most part, winds over 3.3 m/s.

Both *Micasense* multispectral sensors were set to capture images with an intervalometer set at 2 s and automatic exposure. The RGB sensors were typically set on shutter priority with a speed of 1/800th of a second. Due to the slow trigger speed of *Zenmuse X7*, the flight mission speed must be reduced to 3 m/s to ensure a forward overlap of at least 80%.

### Data processing

**Figure 2:**
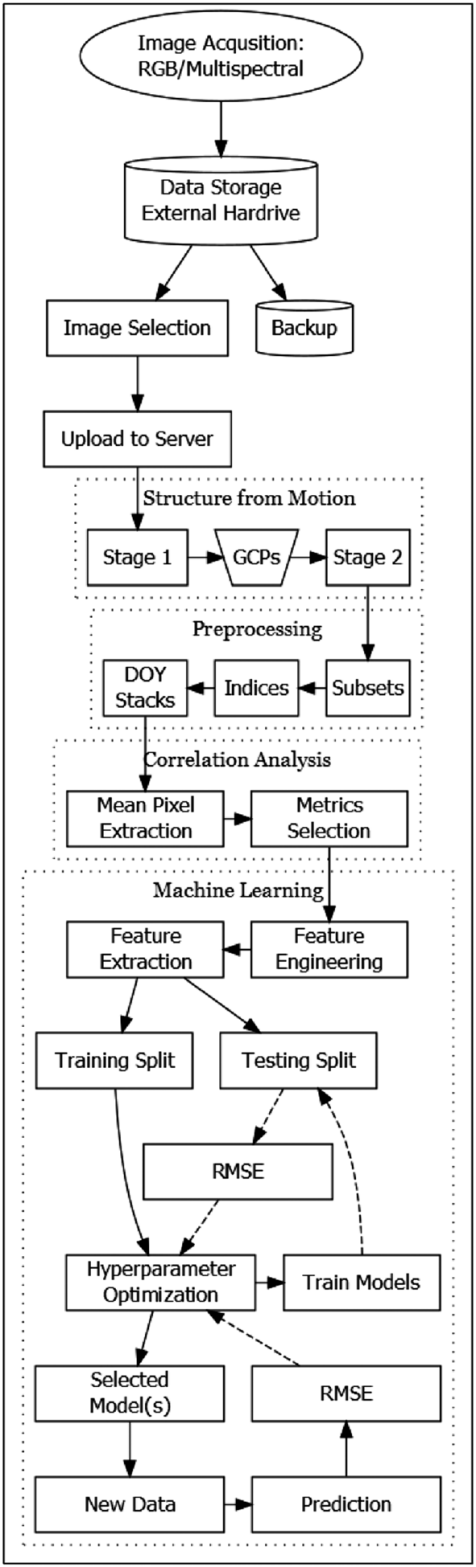
Workflow depicting the various steps from data acquisition to model evaluation.

After each flight campaign, images and metadata were stored on an external hard drive. Naming conventions were based upon a running ID, date, station/district, parcel number, and sensor. After the storage procedure, new similar folder names were created based on individual missions with only the selected images required for the *Structure from Motion* software. These folders were then uploaded to an institute-based Linux High Performance Computing cluster. Photogrammetric products were then produced in *Agisoft Metashape* (v1.7.5) with a semiautomated loop in *Python* where the script was interrupted to manually select ground control points (GCPs) in the RGB and multispectral imagery (see Fig. 2). The GCPs ensured the repeated usage of crown segmentations throughout the time series. The script loops through all folder names per year, which also implemented the naming conventions for each individual photogrammetric product, such as the point cloud, digital surface model (DSM), and orthomosaic.

Further processing was carried out in *R* (R Core Team, 2022), where spectral information was extracted from each individual phenology tree. Each dataset was subset to an area-of-interest with a minimum boundary containing all of the trees used for the ground-based phenological observations. Preceding the calculation of the vegetation indices, the datasets were resampled to 0.01 m for data derived from the RGB sensors and 0.03 m for the multispectral sensors. This enabled layer stacking based on the DOY and sensor. For models that implemented a fusion of both RGB and multispectral data, a resolution of 0.03 m was used. Shapefiles of the manually delineated tree crowns were implemented to extract the mean pixel values from all DOY layer stacks and stored in tabular format for further analysis. For building the models we used training data from 13 to 15 epochs per year. Within each of these epochs multiple phases can be captured, as each separate tree as a phenotype, within a stand, will have varying phase developments at a given time to obtain the largest number of training data for all possible phenological phases. This is necessary to later predict the full time series. Here the main consideration was mapping a tree stand and determine the timing of initial leafing out which gives us information on annual climatic variation as well as potential late frost damage on a stand level. Figure 2 shows the full workflow from image acquisition to model evaluation.

### Vegetation indices

Vegetation indices are widely used in remote sensing and can not only aid in detecting green vegetation traits but also reduce the effects of irradiance and variations in atmospheric transmission^36,37^. Table 4 displays the indices used in this study. The Green Chromatic Coordinate (GCC) and the Normalized Green Red Difference Index (NGRDI) are the two indices that are derived from the visible part of the electromagnetic spectrum and available for typical consumer grade RGB sensors. Indices denoted with a “UC” (e.g., GCC_UC or GNRDI_UC) depict an index derived from bands that did not undergo radiometric calibration (uncalibrated).

**Table 4:** Vegetation indices (VI) used in the study. NIR = Near-infrared.

Name	VI	Formula	References
Green chromatic coordinate	GCC	(Green/red + green + blue)	Gillespie et al.^53^
Normalized green red difference index	NGRDI	(Green–red)/(green + red)	Tucker^54^
Normalized Difference Vegetation Index	NDVI	(NIR–red)/(NIR + red)	Rouse et al.^55^
Normalized Difference Red-edge Red Index	NDRE (ReNDVI)	(Rededge_717_–red_668_)/(Rededge_717_ + red_668_)	Wang^56^
Normalized Difference Red-edge Index	NDREI	(NIR–rededge/(NIR + rededge)	Gitelson and Merzlyak^57^
Green normalized difference vegetation index	GNDVI	(NIR–green)/(NIR + green)	Gitelson et al.^58^
Enhanced vegetation index	EVI	2.5 * (NIR-Red)/(NIR + 6 * red −7.5 * B + 1)	Huete et al.^59^
Normalized difference water index	NDWI	(Green–NIR)/(green + NIR)	McFeeters^60^

The normalized difference red-edge index developed originally by Gitelson and Merzlyak^38^ was labeled NDREI in line with Hunt et al.^39^. The NDREI is sometimes labeled NDRE^40^; however, in this study, for practical reasons, we use the abbreviation NDRE to depict the use of the red-edge and red bands rather than the near-infrared and red-edge bands. Another option could be to use the abbreviation ReNDVI used by Wang^41^.

The air temperature feature (AIRTEMP) was created from a summation of the daily air temperature above 0 °C (“warming days”) since January 1st of a given year and implemented in this study for experimental purposes.

### Feature selection

With the aim of selecting appropriate features for the modeling process, a correlation analysis among independent and dependent variables was implemented. The correlation coefficient is scaleless and represented with the letter *r,* which is interpreted with values between − 1 and 1, where − 1 would represent a perfect negative correlation in that the two variables have an inverse relationship and 1 represents a perfect linear relationship. 0 would depict the situation where both variables have no linear relationship^42^. In this study, the nonparametric Spearman correlation matrix was implemented for each of the indices in relation to the phenological phase and foliation. A test for multicollinearity was carried out that explored the between-variable correlation among predicting features^43^ for the purpose of improving feature selection for multivariate models. The use of polynomial regression models of the first to fifth order were also used to further evaluate individual features during the selection process.

### Statistical analysis and machine learning

The ML regression models implemented in this study included GAMs, boosted GAMs, and gradient boosting machines (GBMs). Model training was conducted using *R’s caret* package^44^. The models were trained with an 80/20 split for training and validation, scaling, and tenfold cross-validation. The ML modeling process was divided into three main stages: (1) The training/validation split applied to the subset variations of the 2019, 2020, and 2021 datasets, followed by testing for correlation and polynomial fitting for feature selection. (2) Models were trained using the combined 2019 and 2020 datasets and then tested with the 2021 dataset. (3) Further testing involved selected models trained with subset variations of the 2019, 2020, and 2021 datasets, which were then tested against new single-epoch datasets acquired from unseen data. The rationale behind using subset variations was to identify which years contribute to error propagation.

The accuracy of the ML regression models in this study was assessed using three metrics: RMSE, mean absolute error (MAE), and R-squared. For interpreting RMSE and MAE values, a phase error is considered functional at values below 1.0 and below 10% for foliation. While the acceptable error margin for operational use has not been conclusively determined, an error below 0.5 for phase and 5% for foliation is deemed similar to, or better than, errors resulting from human observations.

## Results and discussion

### Phenological data historical overview

**Figure 3:**
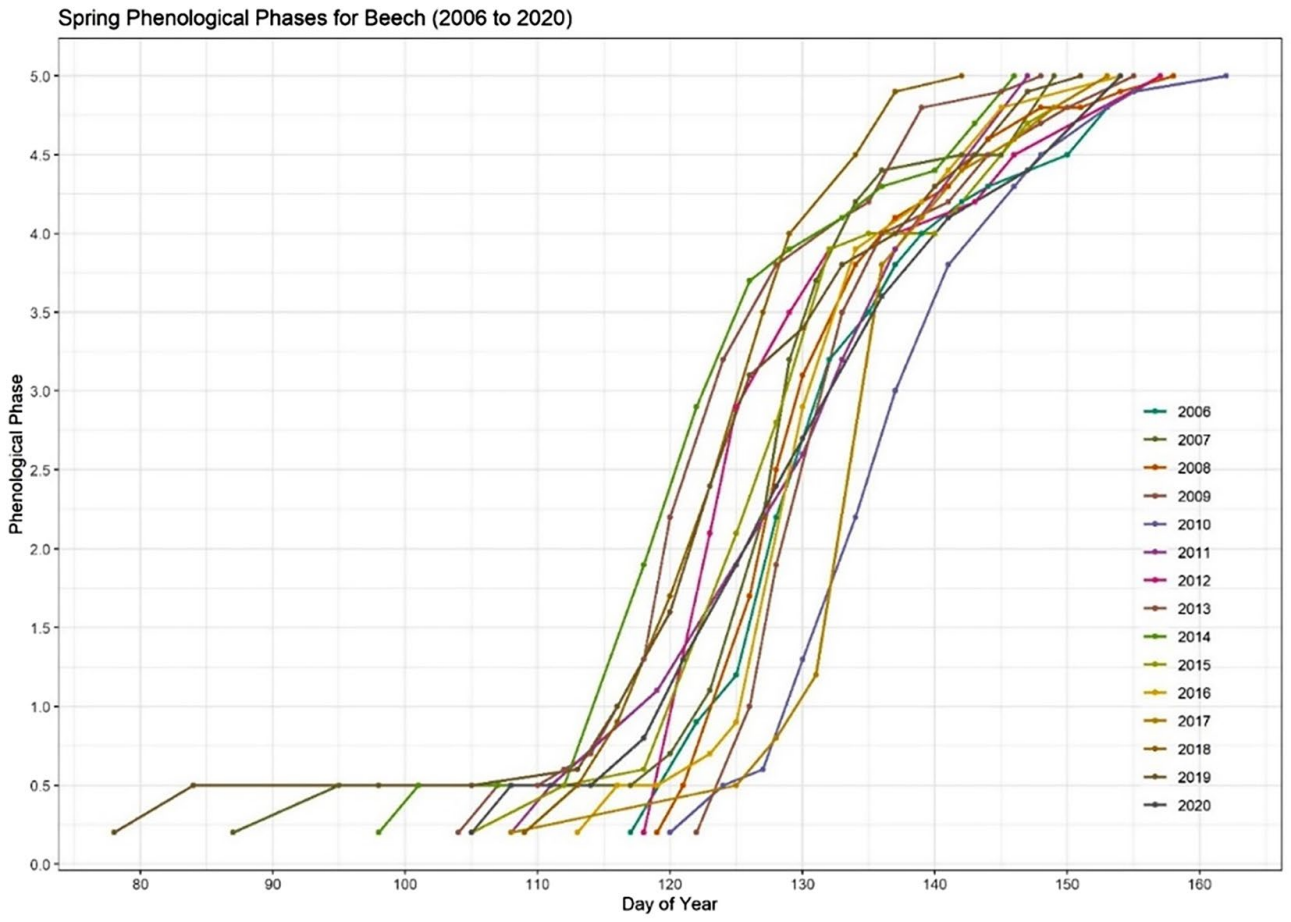
The phenological spring phase development for Beech at the Britz research station between 2006 and 2020. As shown in the figure, the timing of phenological phases can vary considerably over the years, due to a variety of climatic factors.

In the observation period from 2006 to 2020 the process for spring leafing out observations typically began before budburst, at the point when buds began swelling (phase 0.5). Figure 3 shows a comprehensive overview of the yearly averaged spring phenological observations from 2006 to 2020 for the Beech plot with a maximum variation of the start of 40 days and end of all phases of 20 days between the years.

The analysis of the duration between different phenological phases is crucial for understanding two key aspects: first, the timing of budburst in relation to climate change impacts, and second, the progression to later stages, such as phase 4 and phase 5, when leaves are nearing full development. The "hardening" of leaf cell tissues, which occurs at these later stages, renders the leaves less vulnerable to late frosts, intense early spring solar radiation, and biotic pests such as *Orchestes fagi*. Additionally, in early spring drought conditions, certain phases may be delayed, extending the development period from phases 1.0 to 5.0. This phenomenon was observed at the Britz research station in 2006, 2012, 2015, and 2019.

**Figure 4:**
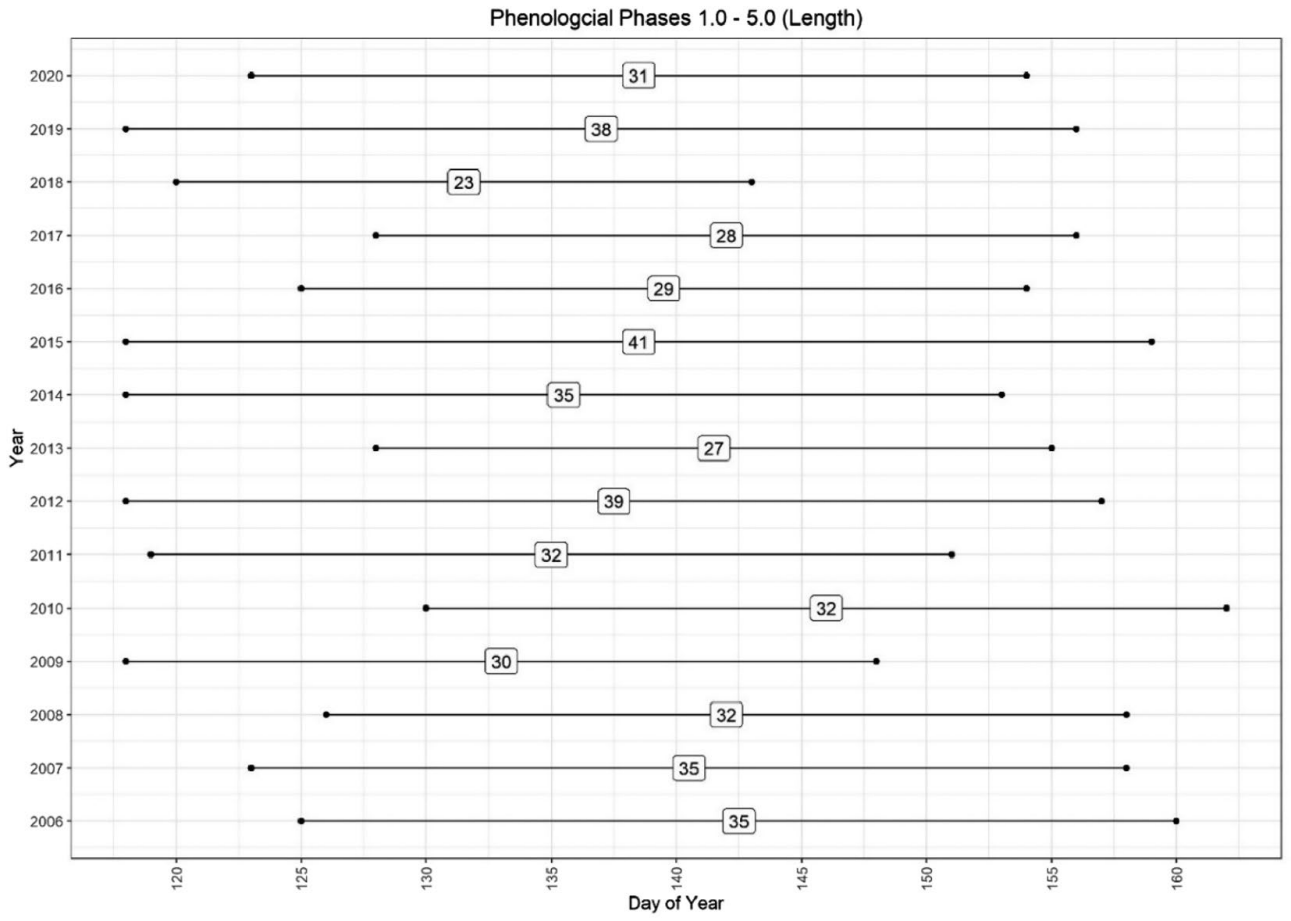
The average spring phenological phases at the Britz research station shown in length between phase 1 and 5 from years 2006 to 2020.

Figure 4 in the study visually illustrates the variability in phase duration from 2006 to 2020, which ranged from 23 to 41 days. Meanwhile, Table 5 offers a comprehensive summary with descriptive statistics for the length of time between phases. The phase lengths presented in Fig. 4 and Table 5 are derived from the average timings across all sampled beech trees in the phenology plot. For more accurate predictions of other phases based on a single observed phase, it might be more effective to model using data from individual trees, given the significant heterogeneity that can exist among them during the spring phenological phases. Further research in this direction is warranted to explore these possibilities.

**Table 5:** An overview of the length between phases from 2006 to 2020. Accuracy is dependent on the temporal resolution of observations.

	2006	2007	2008	2009	2010	2011	2012	2013	2014	2015	2016	2017	2018	2019	2020	Min	Max	Mean	SD
Phase 1–2	4	4	2	2	4	11	6	5	4	8	5	3	3	5	6	2	11	4.8	2.3
Phase 2–3	6	3	4	6	5	5	8	3	5	7	4	5	5	8	7	3	8	5.4	1.6
Phase 3–4	14	13	12	12	11	12	10	9	12	13	8	8	7	14	8	7	14	10.9	2.4
Phase 4–5	11	15	14	10	12	4	15	10	14	13	11	12	8	11	10	4	15	11.3	2.9
Phase 1–4	24	20	18	20	20	28	24	17	21	28	17	16	15	27	21	15	28	21.1	4.3
Phase 1–5	35	35	32	30	32	32	39	27	35	41	29	28	23	38	31	23	41	32.4	4.9

**Figure 5:**
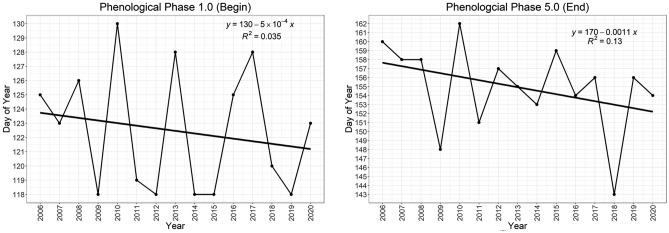
(left) Yearly linear trend in phenological phase 1.0; (right) Yearly linear trend in phenological phase 5.0.

**Figure 6:**
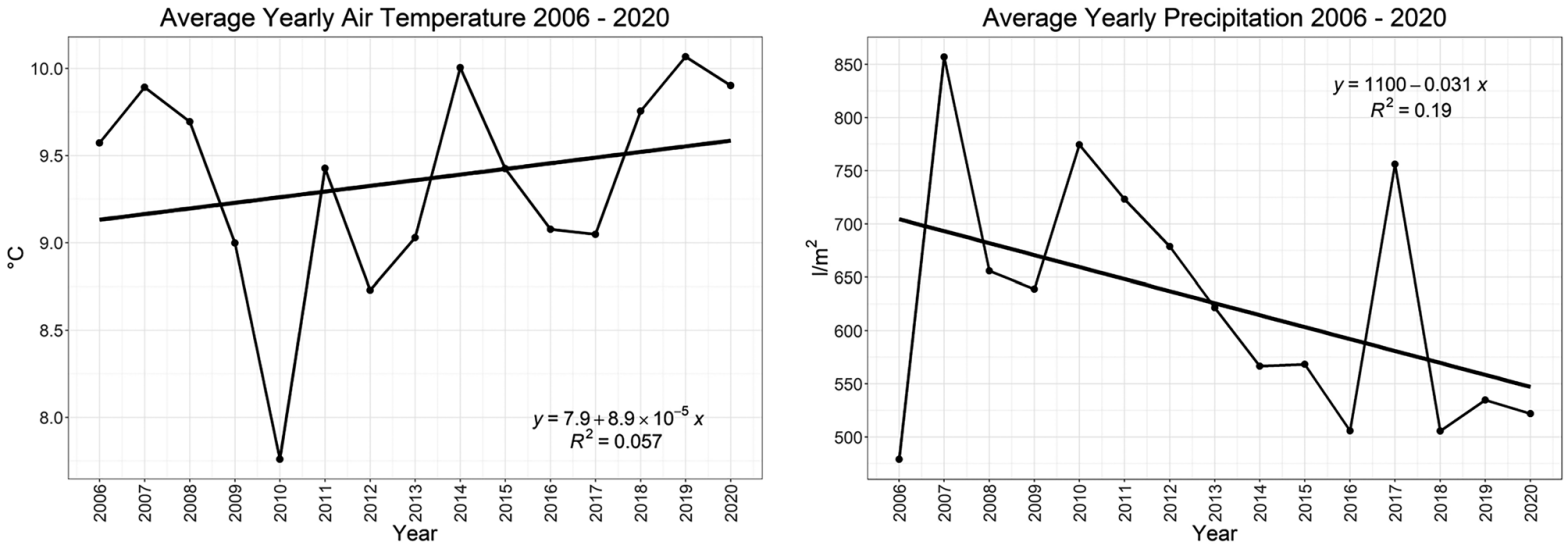
(left) Yearly linear trend of average air temperature between 2006 and 2020; (right) Yearly linear trend of average precipitation between 2006 and 2020. Both are results from the Britz research station.

Trends show an earlier onset of phase 1.0 (see Fig. 5; left), as well as phase 5.0 (see Fig. 5; right). A gradual increase in average yearly air temperature (see Fig. 6; left) is also evident, alongside a steady decrease in yearly precipitation (Fig. 6; right).

**Figure 7:**
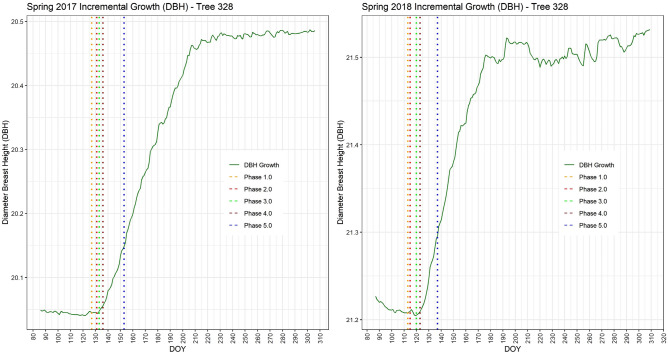
Spring phenological phases shown in relation to band dendrometer measurements from 2017 (left) and 2018 (right). Stem growth typically began around the arrival of phase 3.0.

Several of the trees used for phenological observations at the research site are equipped with electronic band dendrometers and sap flow measurement devices. Figure 7 depicts the relationship between the phenological phases and the onset of stem growth for tree number 328 during the growth season. Notably, in both 2017 and 2018, the onset of stem diameter growth in this tree coincided with the achievement of phase 3.0, which is marked by the emergence of the first fully unfolded leaves.

The dendrometer data from 2018 reveal significant fluctuations in growth deficit throughout the growth season. These fluctuations align with the prolonged drought conditions reported in that year, as documented by Schuldt et al.^45^. This correlation highlights the impact of environmental factors, such as drought, on the growth patterns and phenological development of trees, providing valuable insights into the interplay between climatic conditions and tree physiology.

**Figure 8:**
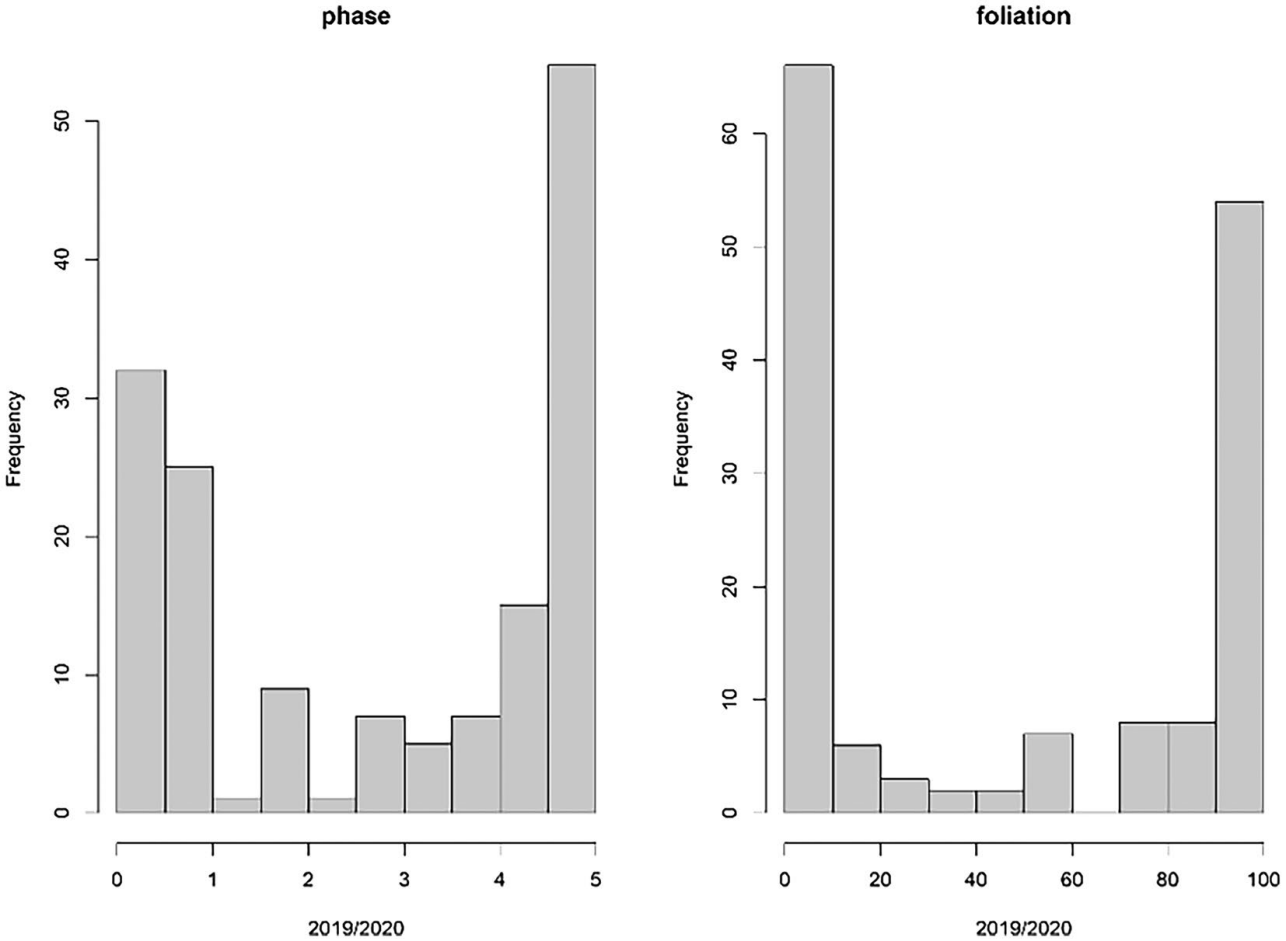
Histograms showing a distinct biomodial distribution of the phase and foliation ground observations from 2019 and 2020.

The analysis of the phase and foliation datasets is further elaborated through the histograms presented in Fig. 8. These histograms exhibit a distinct bimodal distribution, characterized by noticeable left- and right-skewed distributions on the tail ends. This pattern arises from a typical surplus of observations occurring before phase 1.0, which is primarily due to the intensified frequency of observations in anticipation of budburst. Additionally, the extended duration between phases 4.0 and 5.0 contributes to this bimodal distribution. This phenomenon highlights the uneven distribution of observations across different phenological phases, influenced by the varying rates of development and the specific focus of the observation periods.

### Correlation analysis and feature selection

**Figure 9:**
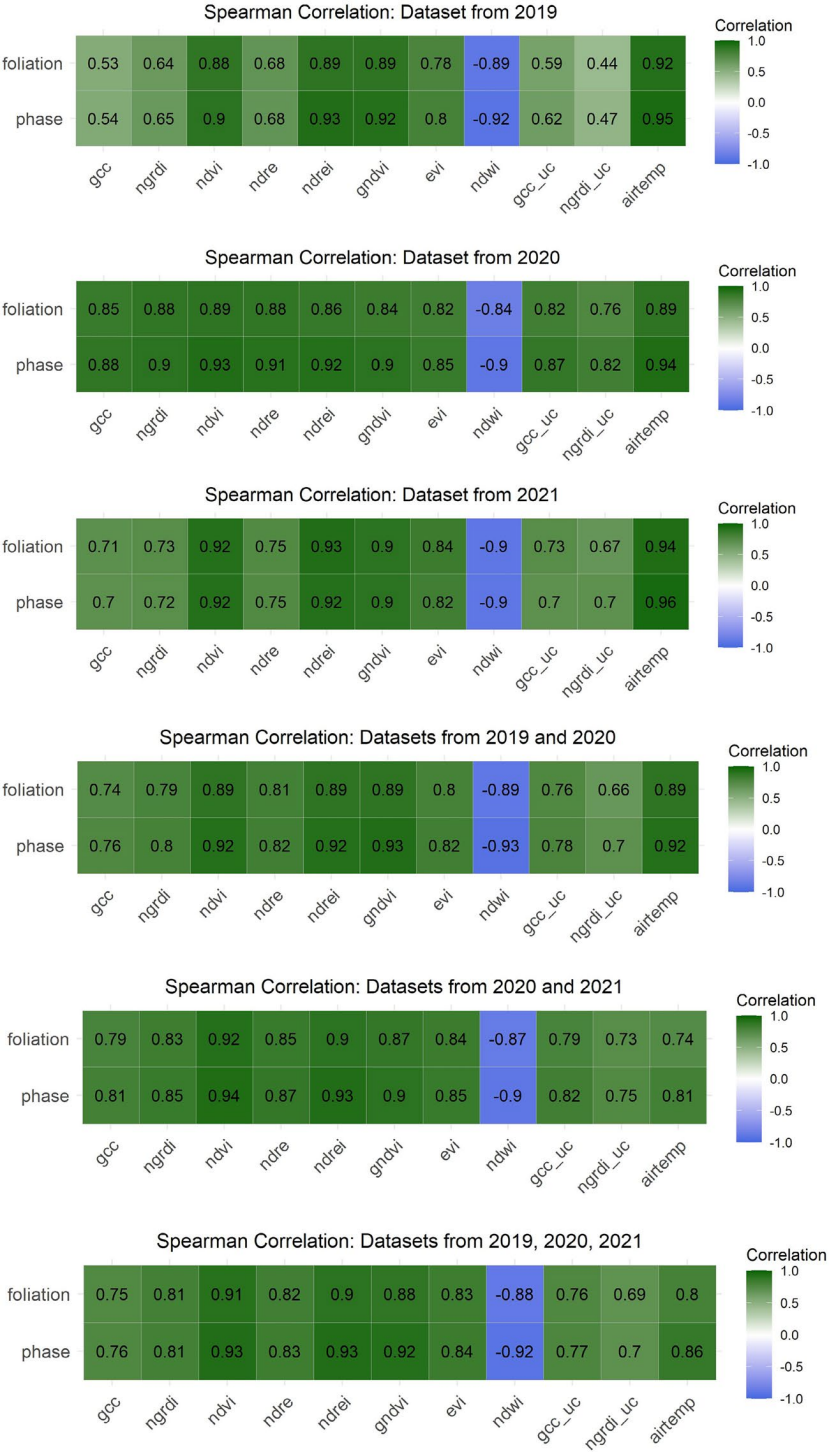
Spearman correlation analysis of the spectral indices derived from the 2019 and 2020 datasets in relation to the ground observations.

Due to the spectral reflectance characteristics of vegetation, visible bands tend to show a positive correlation among each other, whereas the NIR band shows a negative correlation (Mather & Koch, 2011). All the vegetation indices, whether derived from visible or NIR bands or a combination thereof, have a positive correlation with the phase and foliation datasets except for the NDWI, which typically has an inverse relationship with the phases and foliation (see Fig. 9). The most consistent index throughout all datasets, whether originating from single or combined years, is evidently the NDVI with a persistent correlation of *r* > 0.9 (p < 0.001) over all datasets.

Indices derived from visual bands (i.e., GCC and NGRDI) showed a correlation of *r* = 0.65 (p < 0.001), and those uncalibrated were even poorer. Interestingly, the AIRTEMP meteorological-based feature correlated very well with the ground observations (*r* = 0.9; p < 0.001), with a very high correlation coefficient to the phenological phases at *r* = 0.95 (p < 0.001).

**Figure 10:**
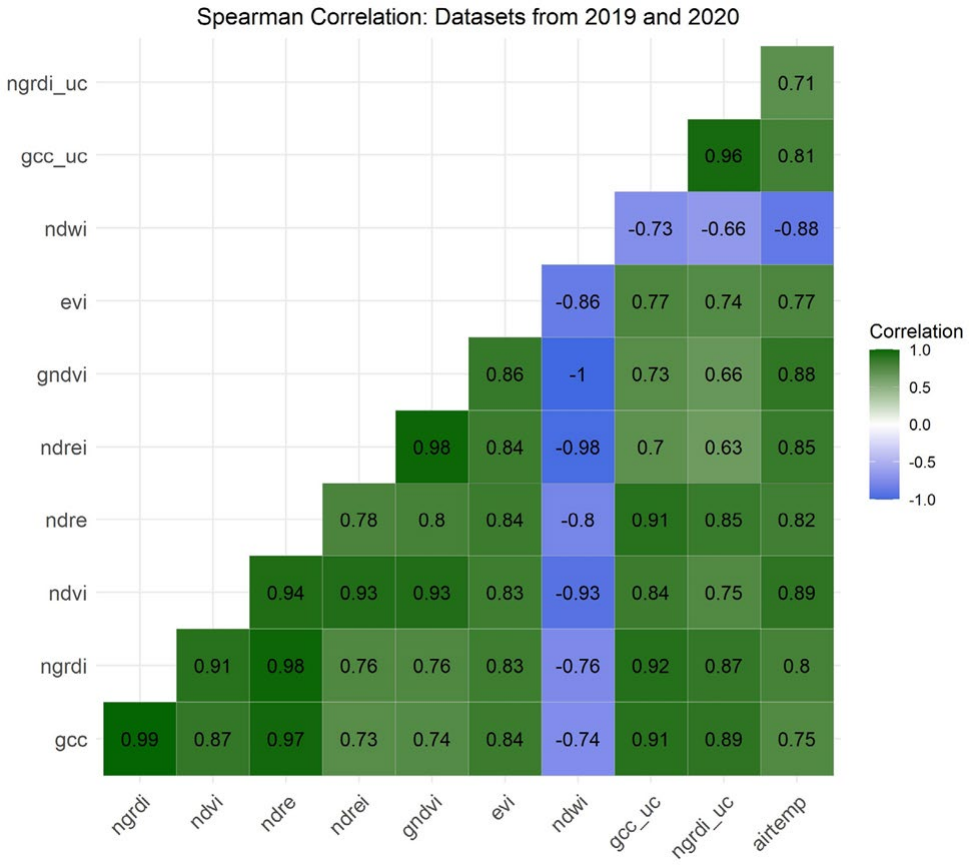
Between-variable Spearman correlation assessment of the 2019/2020 features.

In terms of correlation among independent features (see Fig. 10), the aim was to refrain from implementing highly correlated features when multiple independent features were incorporated into the modeling process. This could be especially problematic when multiple indices are derived from the same bands (i.e., NDVI and EVI). Here, we could deduce that the NDREI and GCC, when used together for the modeling process, have a lower correlation (*r* = 0.73) and do not share any similar bands. Likewise, the NDRE and the NDWI do not share the same bands and have a negative correlation coefficient of *r* = − 0.8. The NDWI and the GCC share only the green band and correlate negatively at *r* = − 0.74.

In analyzing the use of correlation for feature selection, it is important to note that while this method is informative, particularly for evaluating multicollinearity, it can potentially be misleading. This is because correlation coefficients might be artificially high due to the bimodal influence on the dataset. The aggregation of data points at the tail ends of the distribution results in a biased similarity caused by an oversampling of similar phases, thus leading to high correlation coefficients. Consequently, correlation filtering methods were not the sole reliance for feature selection, as outlined by Chandrashekar and Sahin^46^. This approach recognizes the limitations of using correlation analysis in isolation, especially in datasets with unique distribution characteristics such as the one described here.

### Polynomial regression and feature selection

**Figure 11:**
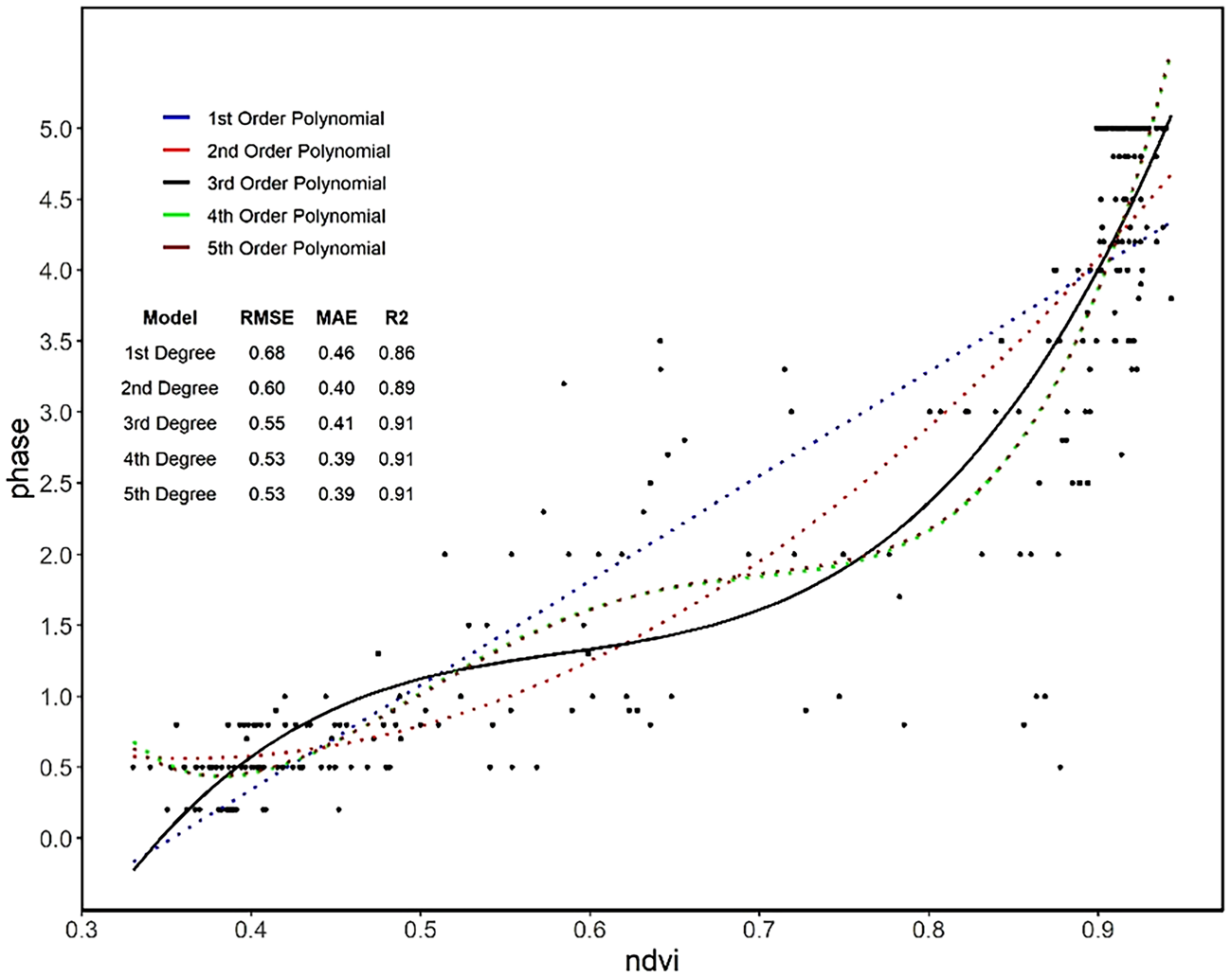
Modelling of the spring phenological phases (2019/2020) dataset with polynomial regression of the first to fifth order.

The addition of polynomial terms into regression models can aid in the characterization of nonlinear patterns^43^ and is conducive to representing phenological trends, particularly those of the spring green-up phases. As polynomial fitting may not be capable of identifying the complexities of phenology metrics in comparison to other algorithms^47,48^, we used the fitting of polynomials here for the purpose of feature selection, where the aim was to identify which features best correspond to the typical spring phenology curve. Figure 11 shows the fitting of the five polynomial orders using the example for the NDVI, resulting in an RMSE of 0.55, MAE of 0.41 and R-squared of 0.91. Here, the third polynomial order was deemed the best choice for further analysis where the curve is not oversimplified or too complex.

**Figure 12:**
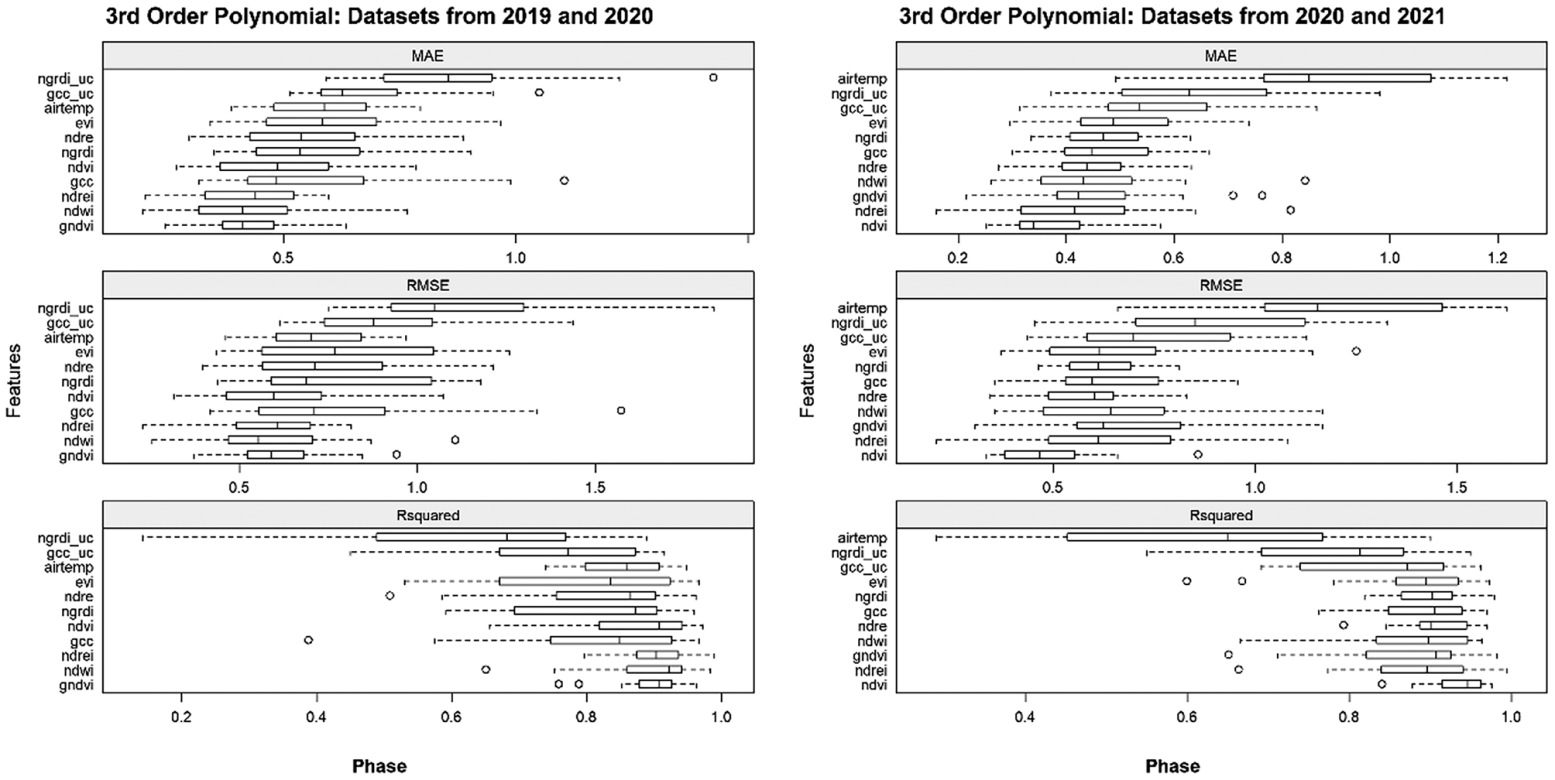
Overview of the spring phenological phases and indices modelled with third-order polynomial regression for the 2019/2020 (left) and 2020/2021 (right) datasets.

**Figure 13:**
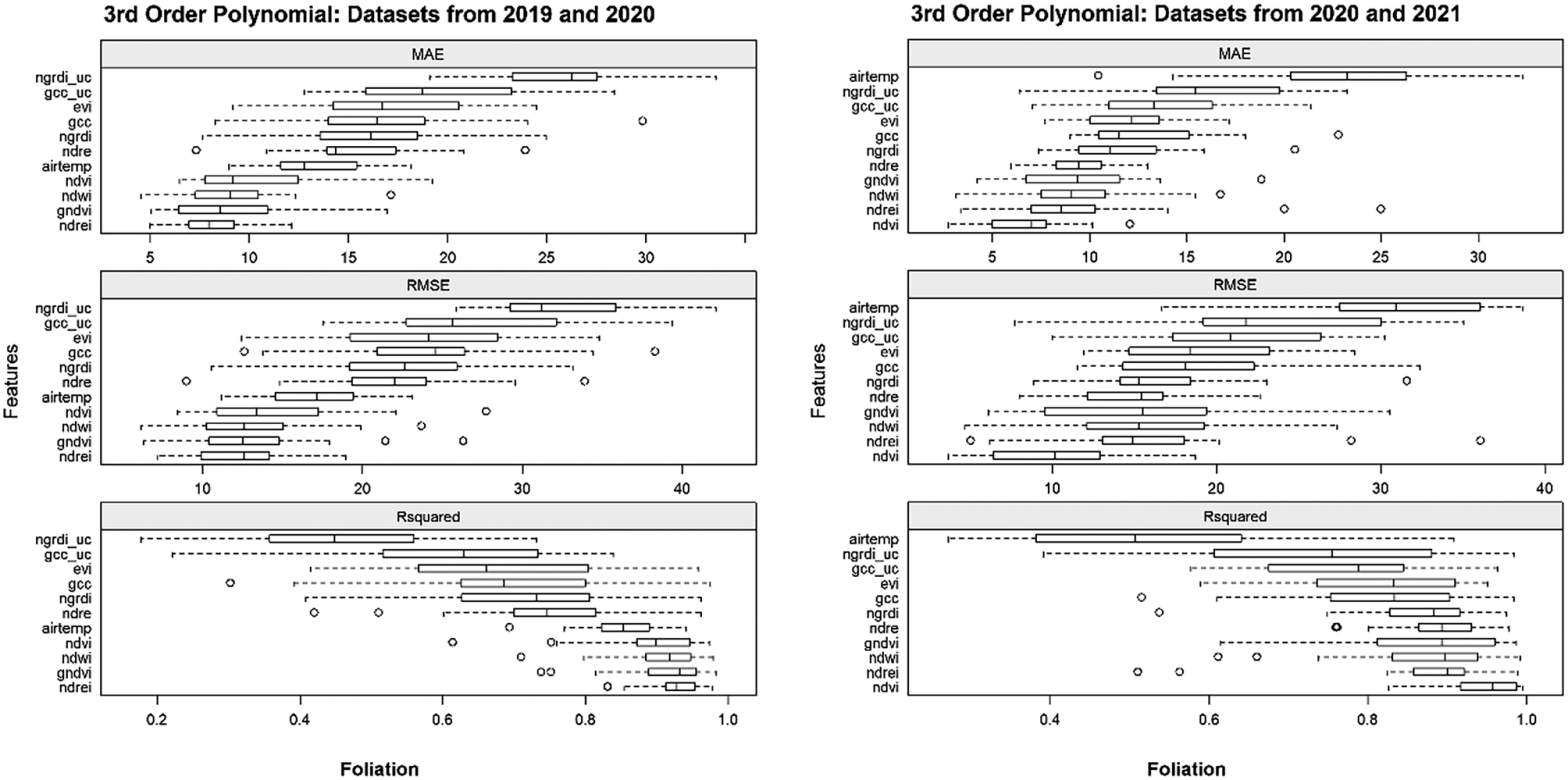
Overview of spring foliation and indices modelled with polynomial regression of the third order for the 2019/2020 (left) and 2020/2021 (right) datasets.

To follow, each of the selected individual features was tested with the 3rd-order polynomial separately for the 2019/2020 and 2020/2021 datasets for both phase (Fig. 12) and foliation (Fig. 13). In terms of the phenological phases, the GNDVI shows quite a low dispersal of RMSE for the 2019/2020 dataset, yet the dispersal is higher for the 2020/2021 dataset. A similar result is evident for the NDVI, where less dispersal is found in the 2020/2021 dataset than in the 2019/2020 dataset. The cumulative warming days (AIRTEMP) as well as the indices derived from the uncalibrated visible bands (GCC_UC and NGRDI_UC) fared poorly for both datasets. This was also the case for foliation; however, AIRTEMP performed better for the 2019/2020 dataset. Regarding foliation, the NDVI also performed well for the 2020/2021 dataset, as did the NDREI for both datasets.

### Machine learning models: 2019/2020 datasets

Based on the results of the correlation analysis and polynomial fitting, we were able to select the most relevant features for further scrutinization during the subsequent modeling process. It is important to note here that in the initial feature selection process using only the correlation analysis alone could have produced an unseen bias due to an aggregation of data points at the tail ends of the datasets, which was especially evident for the 2019/2020 dataset. We proceeded to build three models based on ML algorithms that aided in choosing the best performing algorithms as well as features. Each of the selected individual and combined indices were modeled with each algorithm and evaluated using an 80/20 training/validation data split. This not only helped in choosing the best ML algorithm but also assisted in a type of model-based feature selection by further narrowing down the selected features. In terms of the phenological phases, an RMSE of ≤ 0.5 (0.6) is deemed acceptable and similar to the magnitude of potential human error. For the Britz method of foliation, an RMSE of ≤ 10% is assumed to be acceptable; however, some may argue that an RMSE of ≤ 5% in terms of foliage observations is possible with ground observations. Here, it should be noted that the Britz method of foliation is based on the percentage of leaves that have fully opened rather than fractional cover or greening-up.

### Phenological phases

Regarding the phenological phases, the GAM boosting algorithm showed the best results overall (see Table 6). The GAM models with the features NDREI + GCC resulted in an RMSE of 0.51, MAE of 0.33 and an R-squared of 0.95. The feature combination of NDWI + GCC resulted in an RMSE of 0.46, MAE of 0.3 and R-squared of 0.96. The top performing model was that of GAM boosting with the NDVI, which produced an RMSE of 0.28, MAE of 0.18, and R-squared of 0.98. The second-best performing model was that of the GAM model with the NDRE + NDWI input features, resulting in an RMSE of 0.44, MAE of 0.31 and R-squared of 0.96. Interestingly, the uncalibrated GCC (GCC_UC) outperformed the calibrated GCC with an RMSE of 0.73 for gradient boosting and the GCC_UC index as opposed to an RMSE of 0.81 for GAM boosting and the GCC.

**Table 6:** Error metrics for the phase prediction of three machine learning algorithms. Values shown in bold font depict the best results.

Phases	GAM			GAM boosting			Gradient boosting		
	RMSE	MAE	R^2^	RMSE	MAE	R^2^	RMSE	MAE	R^2^
GNDVI	0.81	0.5	0.87	0.8	0.49	0.87	0.82	0.5	0.86
NDREI	0.75	0.43	0.89	0.75	0.41	0.89	0.78	0.46	0.89
NDVI	0.32	0.2	0.98	0.28	0.18	0.98	0.38	0.3	0.98
NDRE	0.65	0.53	0.95	0.64	0.53	0.95	0.66	0.58	0.98
GCC	0.84	0.64	0.9	0.81	0.63	0.90	0.82	0.64	0.90
NDREI + GCC	0.55	0.37	0.9	0.51	0.33	0.95	0.6	0.39	0.93
NDWI + GCC	0.48	0.35	0.96	0.46	0.3	0.96	0.49	0.33	0.96
NDRE + NDWI	0.44	0.31	0.96	0.5	0.33	0.95	0.47	0.33	0.96
GCC_UC	0.76	0.62	0.93	0.75	0.62	0.93	0.73	0.62	0.96

At this stage of the modeling process, the NDVI and GAM boosting algorithms showed very good results (RMSE = 0.28), and the question is here whether the dataset is overfit for the Britz research station beech stand (Table 7). At this point, it is imperative to test the models with unseen data and assess which ones are generalizable over various beech stands, especially those of increased age. In terms of the models derived from indices from the visual bands, the uncalibrated GCC performed slightly better than the radiometrically calibrated GCC and better than some of the models derived from the calibrated multispectral bands, which is particularly interesting, as RGB sensors are typically acquired at a much cheaper price.

**Table 7:** Error metrics (in %) for the foliation prediction of three machine learning algorithms. Values shown in bold font depict the best results.

Foliation	GAM			GAM boosting			Gradient boosting		
	RMSE	MAE	R^2^	RMSE	MAE	R^2^	RMSE	MAE	R^2^
GNDVI	22	10	0.83	22	10	0.83	22	11	0.8
NDREI	21	11	0.84	21	11	0.84	21	10	0.85
NDVI	7	4	0.98	7	4	0.98	9	6	0.97
NDRE	16	12	0.97	16	12	0.97	16	12	0.97
GCC	22	16	0.91	22	16	0.91	22	16	0.92
NDREI + GCC	17	9	0.90	17	9	0.91	16	8	0.91
NDWI + GCC	13	7	0.94	13	7	0.94	14	7	0.94
NDRE + NDWI	14	8	0.93	14	8	0.93	14	7	0.93
GCC_UC	21	16	0.96	21	16	0.96	21	17	0.97

### Foliation

For the most part, all models failed the 10% cutoff point except for those using the NDVI as an input feature. Both the NDVI-based GAM boosting and gradient boosting models obtained an RMSE of 7%, MAE of 4% and R-squared of 0.98. Here, overfitting could also be a factor; however, it will still be interesting for further model assessment of the prediction of foliation on a new dataset (2022) as well as datasets outside of the Britz research station. The worst performing models were those utilizing the radiometrically calibrated GCC, which acquired an RMSE of 22%, MAE of 16%, and R-squared of 0.92.

### GAM boosting models with test datasets

With the aim of testing the robustness and generalizability of the developed models, new data from 2022 as well as data from different forest stands (beech) were introduced (Table 7). Here, we tested the models on new spring phenological data from the same stand from 2022 (*n* = 17) as well as an older beech stand in Kahlenberg (*n* = 10) located in the same region as the Britz research station and a beech stand in the more mountainous region of the Black Forest (*n* = 8) in southwestern Germany. The three test datasets are limited to only one Epoch, where the Kahlenberg site is comprised of mostly later phases and the Britz and Black Forest datasets have a wide range of earlier phases (< 4.0). Additionally, training datasets were divided into three different subdivisions based on the year of origin: 2019/2020, 2020/2021 and all datasets together (2019–2021). This was carried out for the purpose of distinguishing whether data acquisition methods from a certain year contributed to error propagation. For example, the 2019 field data were collected by a different observer and often not recorded on the same day as flights (± 3 days), as well as low-quality radiometric calibration. The models chosen for testing were those implementing GAM boosting and the RGB-derived indices GCC (Micasense Altum) and GCC_UC (Zenmuse X7) and the NDVI (Micasense Altum). Table 8 displays a list of all the tested models with reference to the applied index, location, training data subdivision and date.

**Table 8:** displays the various models tested on the datasets from 2022 and/or outside of the Britz research station.

Model Name	Site	Index	Training	DOY	Year
br-ndvi-19–20	Britz	ndvi	2019–2020	125	2022
**br-ndvi-20–21**	**Britz**	**ndvi**	**2020–2021**	**125**	**2022**
br-ndvi-19–21	Britz	ndvi	2019–2021	125	2022
br-gcc-19–20	Britz	gcc	2019–2020	125	2022
**br-gcc-20–21**	**Britz**	**gcc**	**2020–2021**	**125**	**2022**
br-gcc-19–21	Britz	gcc	2019–2021	125	2022
br-gcc-uc-19–20	Britz	gcc_uc	2019–2020	125	2022
br-gcc-uc-20–21	Britz	gcc_uc	2020–2021	125	2022
br-gcc-uc-19–21	Britz	gcc_uc	2019–2021	125	2022
bf-gcc-19–20	Black forest	gcc	2019–2020	115	2022
**bf-gcc-20–21**	**Black forest**	**gcc**	**2020–2021**	**115**	**2022**
bf-gcc-19–21	Black forest	gcc	2019–2021	115	2022
bf-gcc-uc-19–20	Black forest	gcc_uc	2019–2020	115	2022
bf-gcc-uc-20–21	Black forest	gcc_uc	2020–2021	115	2022
bf-gcc-uc-19–21	Black forest	gcc_uc	2019–2021	115	2022
ka-ndvi-19–20	Kahlenberg	ndvi	2019–2020	119	2022
ka-ndvi-20–21	Kahlenberg	ndvi	2020–2021	119	2022
ka-ndvi-19–21	Kahlenberg	ndvi	2019–2021	119	2022
ka-gcc-19–20	Kahlenberg	gcc	2019–2020	135	2022
ka-gcc-20–21	Kahlenberg	gcc	2020–2021	135	2022
ka-gcc-19–21	Kahlenberg	gcc	2019–2021	135	2022
ka-gcc-uc-19–20	Kahlenberg	gcc_uc	2019–2020	135	2022
**ka-gcc-uc-20–21**	**Kahlenberg**	**gcc_uc**	**2020–2021**	**135**	**2022**
ka-gcc-uc-19–21	Kahlenberg	gcc_uc	2019–2021	135	2022

The four models in bold font are those deemed operational.

**Figure 14:**
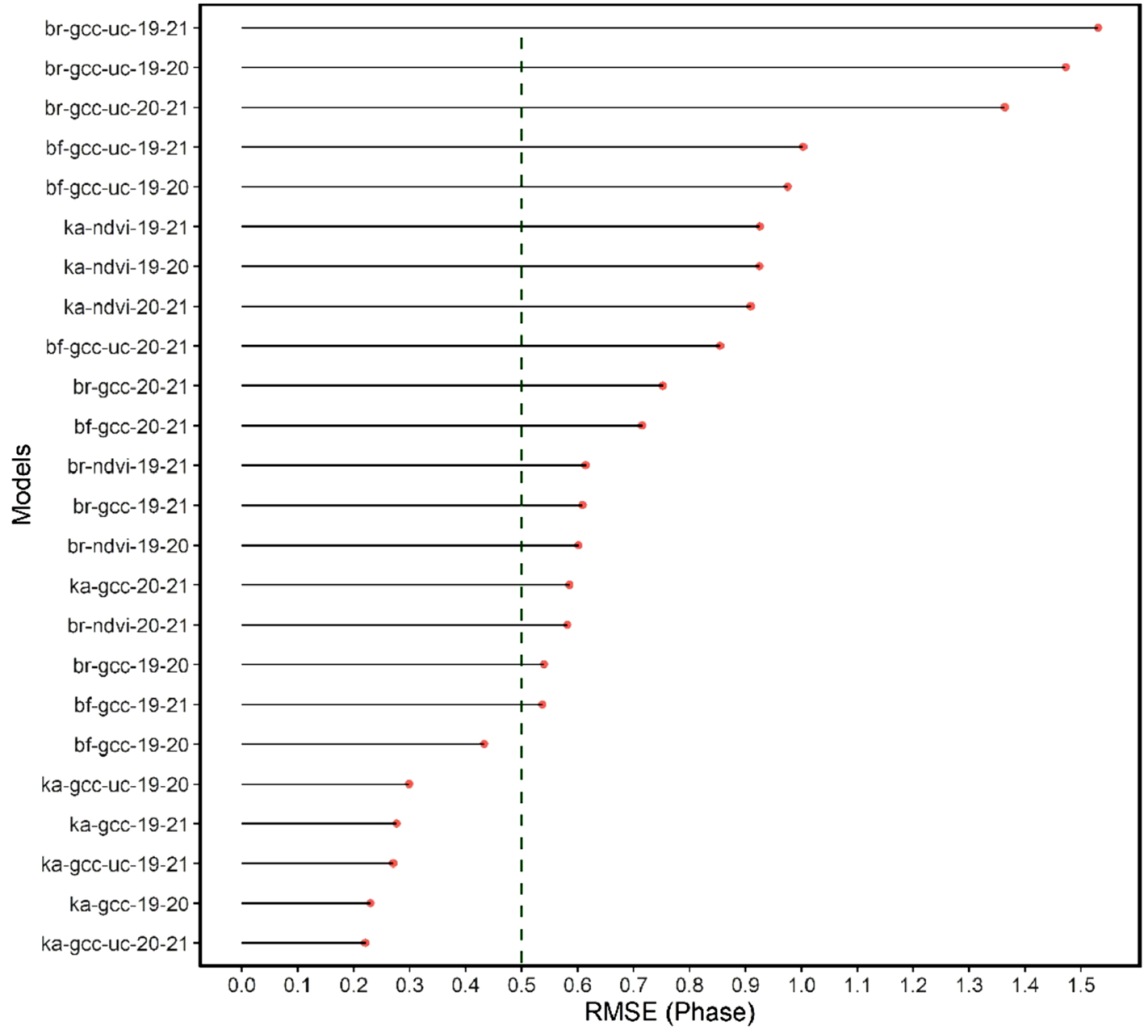
graph showing the RMSE for the phase prediction ranked in order from poorest to best RMSE. The green dashed line depicts the cut-off point of acceptable accuracy. Allowing an RMSE of up to 0.6 would enable the NDVI model derived from the multispectral datasets. Otherwise, only models originating from the visible bands are considered operational.

**Figure 15:**
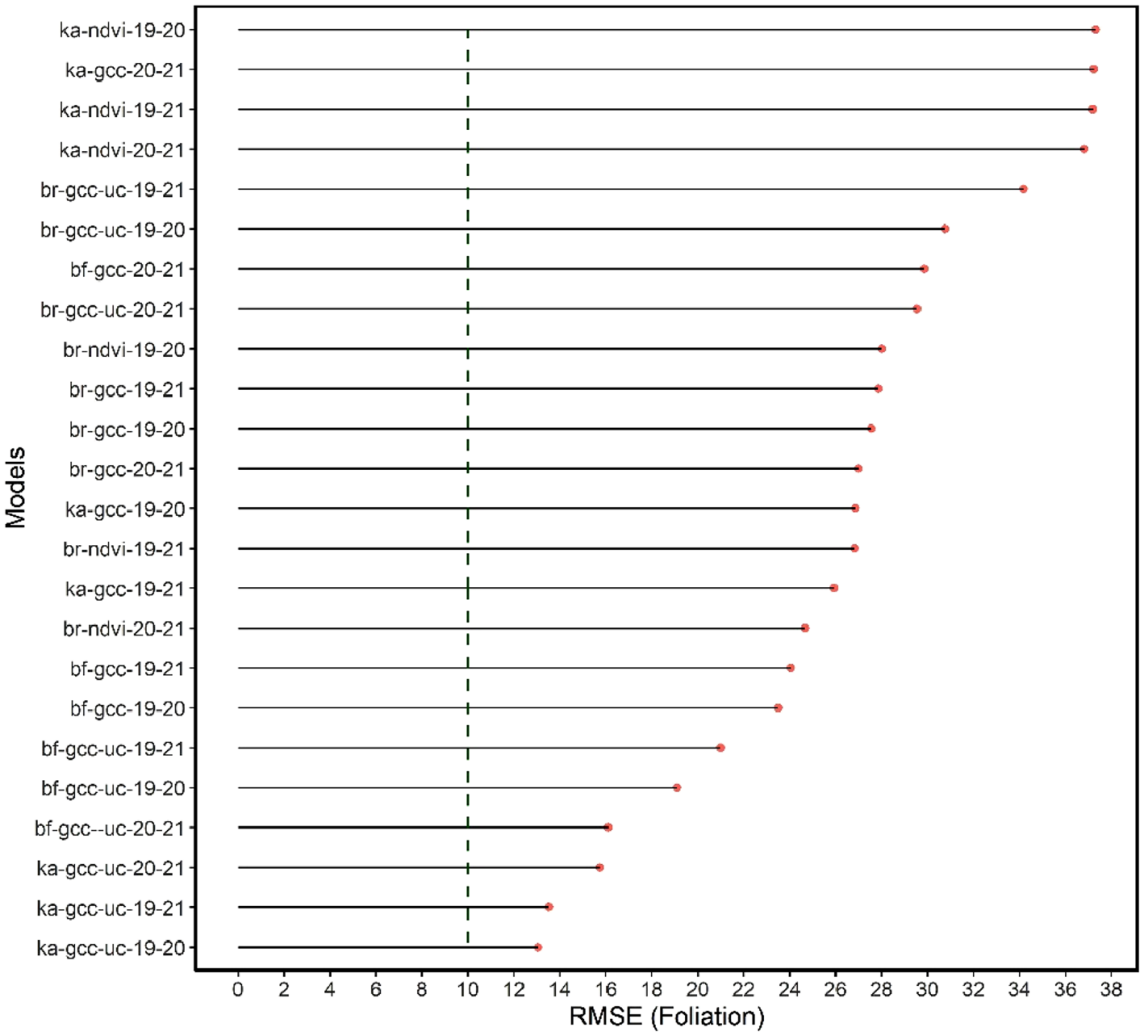
graph showing the RMSE for foliation prediction ranked in order from poorest to best. The green dashed line depicts the cut-off point of 10%. None of the models for foliation prediction are considered functional.

**Figure 16:**
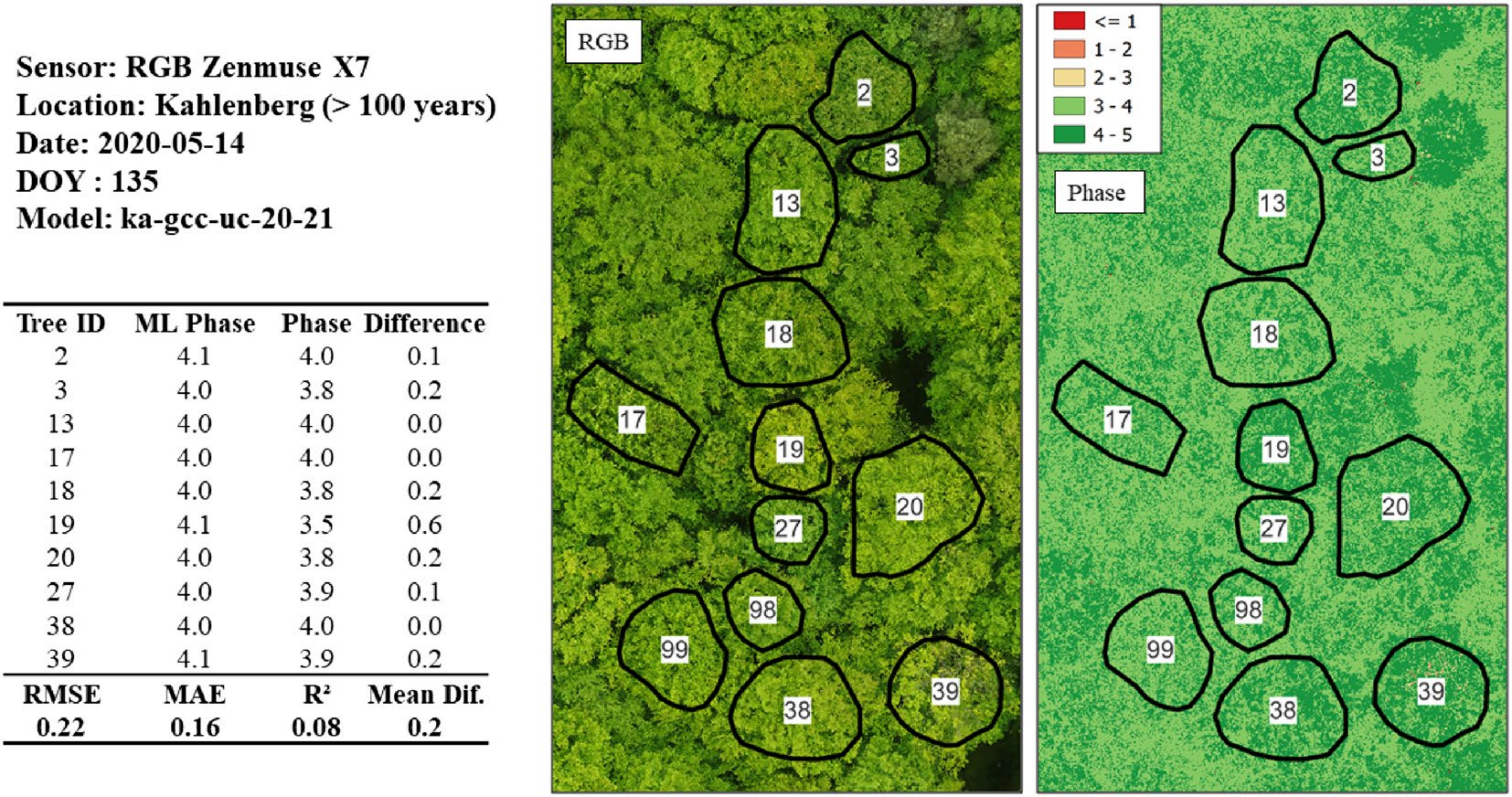
Phase prediction of an older beech stand (> 100 years) utilizing the model originating from the uncalibrated GCC 2020/2021 dataset. The very low RMSE of 0.22 proves a highly generalizable model; however, it should be noted that this is a relatively small dataset (n = 10) and comprised of only later phases (> 3.0). The “ML phase” is the predicted phase, and the “Phase” originates from ground-based observations.

**Figure 17:**
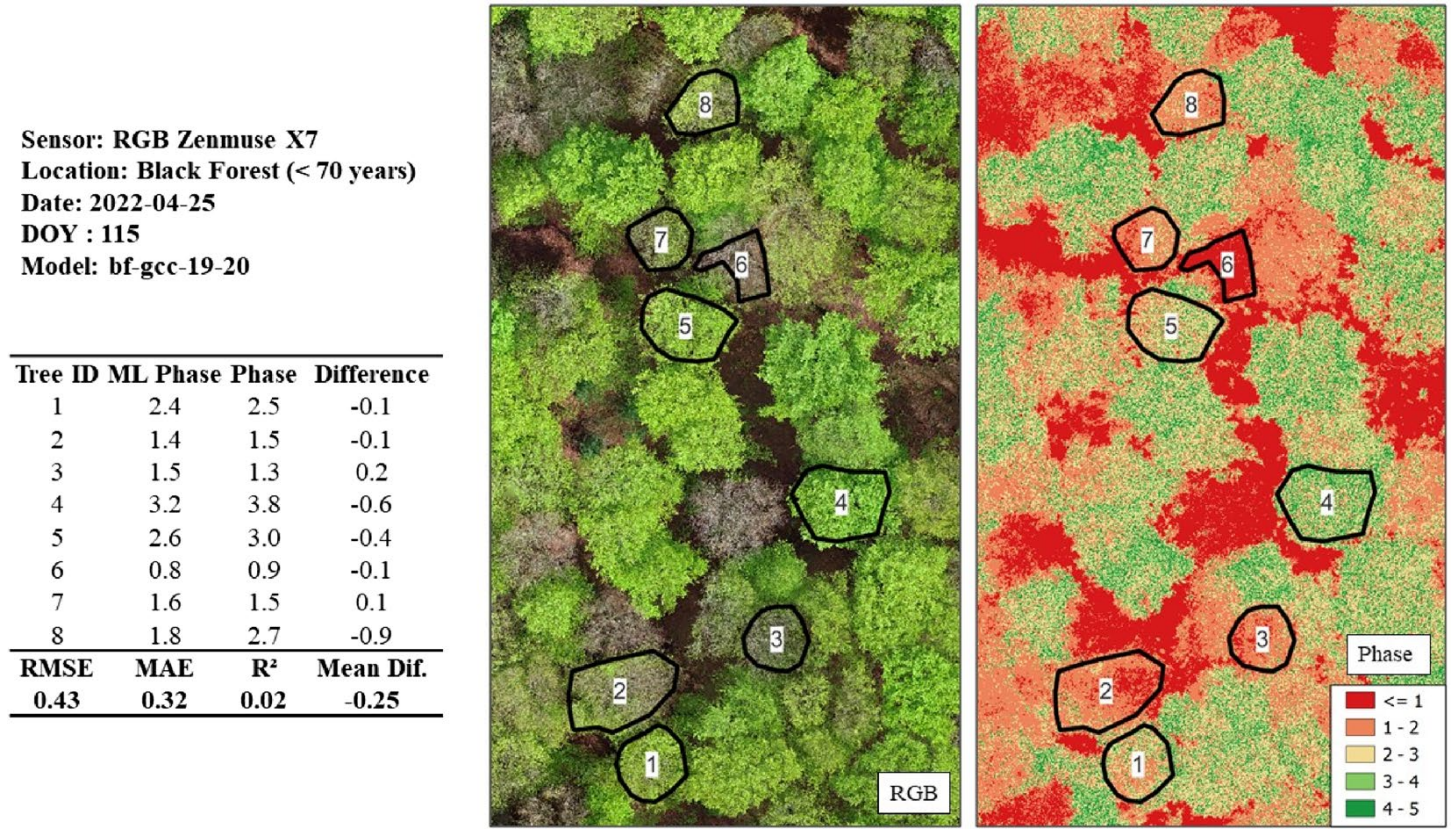
Phase prediction of a beech stand (< 70 years) utilizing the model originating from the calibrated GCC 2019/2020 dataset. The Black Forest dataset is particularly challenging, as a wide range of phases are available. An RMSE of 0.43 is within the accepted error cut-off of ≤ 0.5.

**Figure 18:**
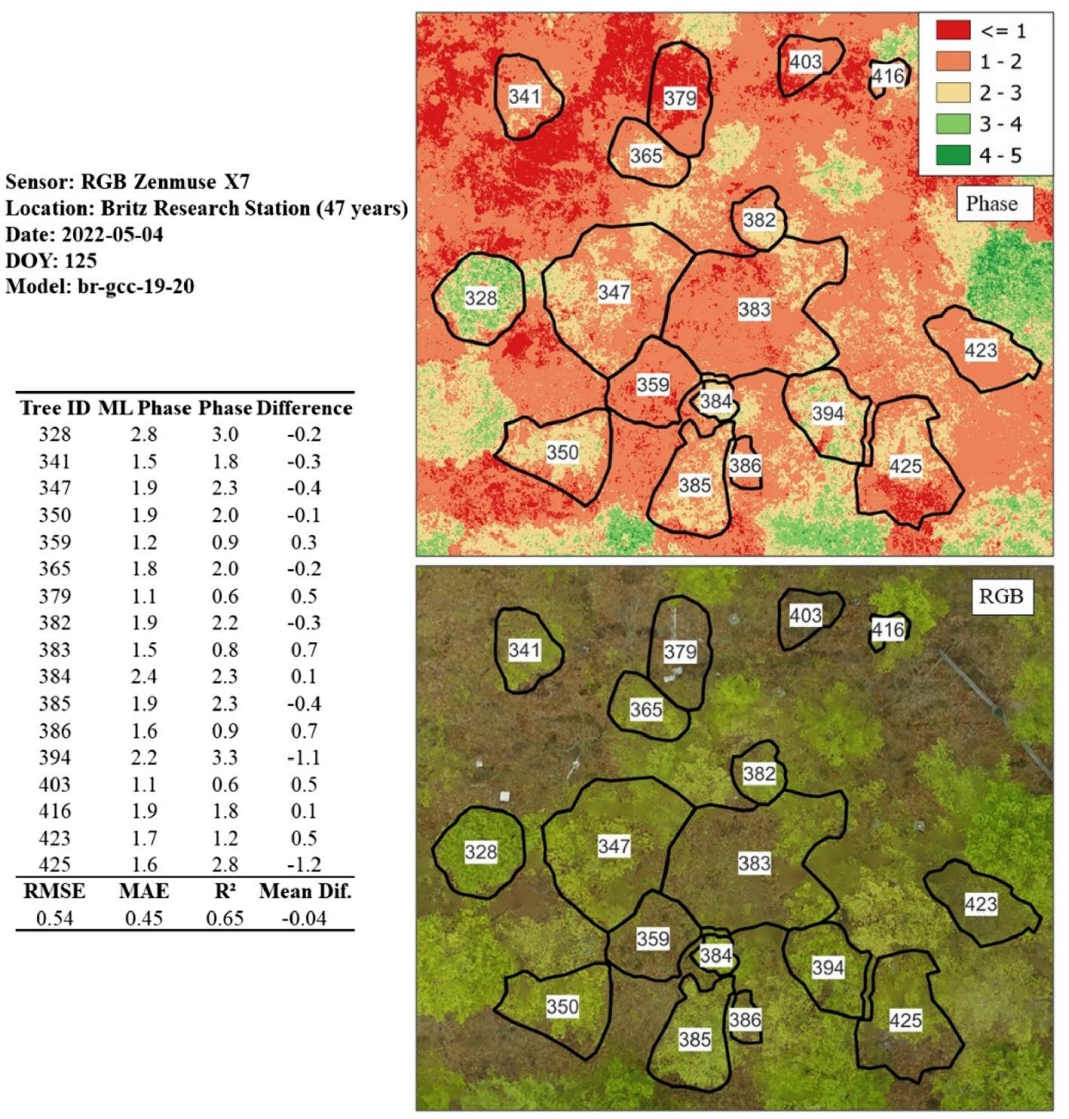
Phase prediction of a beech stand (47 years) utilizing the model originating from the calibrated GCC 2020/2021 dataset. Despite being a larger dataset (n = 17) in comparison to the other test sites, an RMSE of 0.54 was achieved, which can be regarded as achieving the 0.5 threshold.

**Figure 19:**
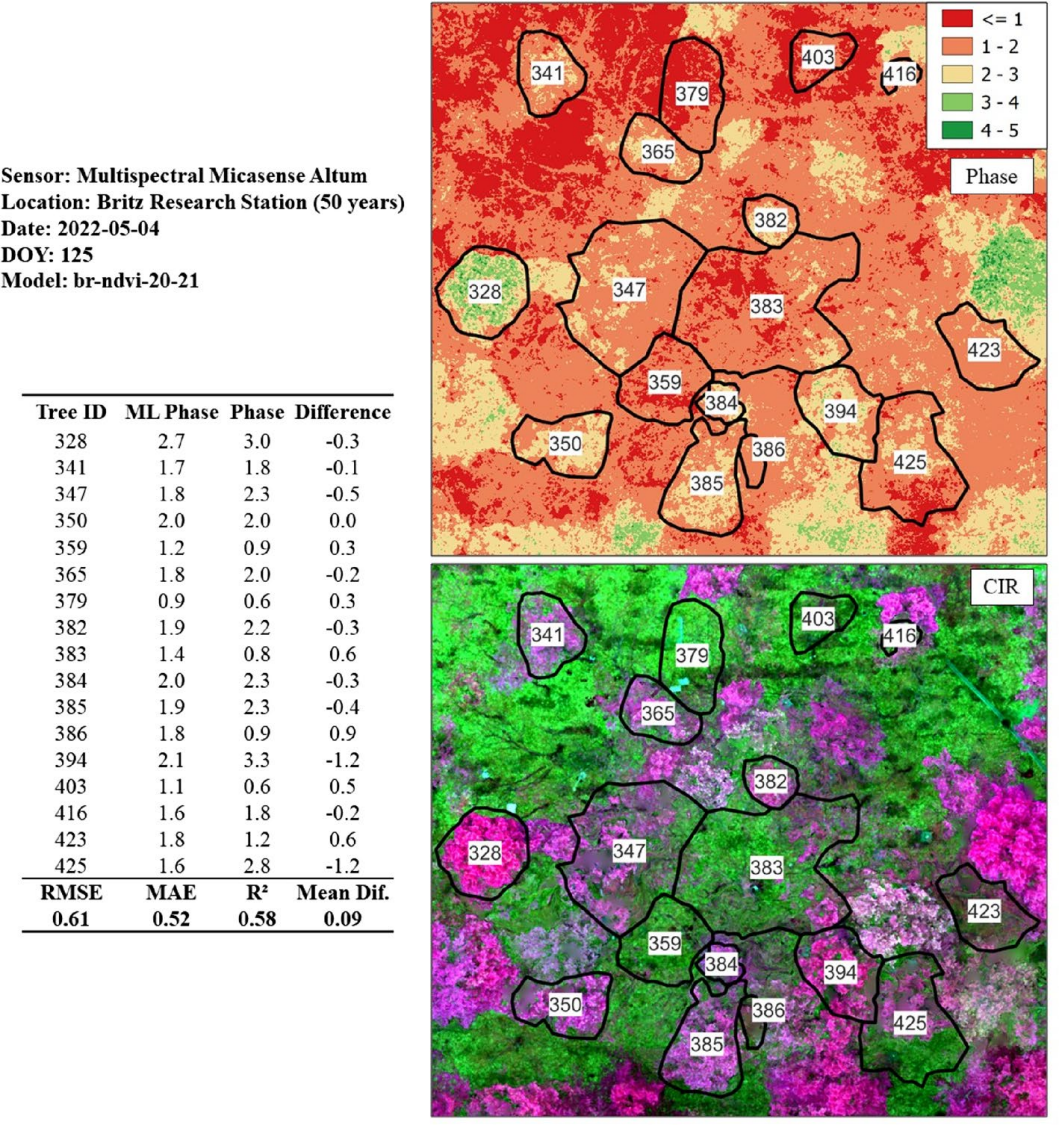
Phase prediction of a beech stand (50 years) utilizing the model originating from the calibrated NDVI 2020/2021 dataset. This is the only model derived from the nonvisible band (NIR), which is in proximity to the 0.5 threshold RMSE = 0.61). CIR = Color-infrared.

The results of the model testing of the phenological phase prediction (see Fig. 14) and foliation (see Fig. 15) were ranked in order of the RMSE. Notably, all the models of the phenological phase prediction that achieved the 0.5 threshold (left of green dotted line) were those of the calibrated and uncalibrated GCC, which originate from bands of the visible portion of the electromagnetic spectrum. Five of six of these models were from the Kahlenberg dataset, and one was from the Black Forest dataset. The best performing models were selected for each of the test sites and are mapped out in Figs. 16, 17, 18, 19. All image data acquired for the test sites with *Zenmuse X7* lack radiometric calibration except for the Britz dataset (see Fig. 19), which was acquired with both the *X7* and radiometrically calibrated *Micasense Altum* data.

The Kahlenberg dataset (see Fig. 16) with the *gcc-uc-20–21* model resulted in a very low RMSE of 0.22, MAE of 0.16 and R-squared of 0.08 (*n* = 10). Such a low RMSE for an uncalibrated RGB-based model is an unexpected result here and shows that the later phases, in particular phase 4.0, predict well. Phase 4.0 is a significant phase in the spring green-up, as it corresponds to the completion of all leaf and shoot development. The transition to Phase 5.0 would then follow with the hardening of leaf tissue alongside a change to darker green and increased late-frost hardiness.

Regarding the Black Forest dataset with the *bf-gcc-19-20* model, an RMSE of 0.43, MAE of 0.32, and R-squared of 0.02 (*n* = 8) were achieved (see Fig. 17). Here, a scene with a wide range of phases (0.9–3.8) was available, and a successful phenological phase prediction was possible with the calibrated GCC model and training data from 2019 and 2020. It is important to note that the radiometrically calibrated GCC model was used to predict the GCC, which is derived from the noncalibrated *Zenmuse X7*. Significant here is that sensor mixing in terms of model training with the multispectral sensor and prediction with a consumer grade RGB sensor is attainable. We considered the low R-squared as insignificant due to the overall low sample rate of the test datasets.

The Britz dataset (see Fig. 18) also implemented the GCC and 2019/2020 training model (*br-gcc-19-20)* and resulted in an RMSE of 0.54, MAE of 0.45 and R-squared of 0.65 (*n* = 17). It is important to note that the Britz test dataset possesses more samples than other test sites and achieves the 0.5 threshold. This test dataset, however, comprises the same trees as those in the training dataset, providing the model with an advantage at the Britz test site. It is important to note, however, that this advantage might not extend to other test sites, potentially limiting the model's ability to generalize well in different settings.

With respect to the test sites involving phase prediction from the multispectral sensor (*Micasesense Altum*), only the Britz and Kahlenberg sites were available. The only NDVI-based model that was in proximity to the 0.5 threshold was the Britz test dataset (*br-ndvi-20-21),* with an RMSE of 0.61, MAE of 0.52, and R-squared of 0.58 (*n* = 17). We hypothesized that the radiometric calibration methods from 2019 would influence the model accuracy; however, there was only a marginal difference in the RMSEs of the 2019/2020 and 2020/21 datasets.

Overall, the best performing and most consistent model for predicting the spring phenological phases was the calibrated GCC model trained on the 2019/2020 dataset. This model (gcc-uc-19-20) demonstrated strong generalization across all test sites, including the Black Forest (bf-gcc-19-20) and Kahlenberg (ka-gcc-uc-19-20), with the highest RMSE observed at the Britz (br-gcc-uc-19-20) 2022 test site (RMSE = 0.54). For a visual representation of the model's performance, please refer back to Fig. 14.

### Sources of error and synopsis

This research highlights the challenges in obtaining radiometrically calibrated datasets over multiple growing seasons, despite pre- and post-mission calibration panel acquisition and DLS data usage. Issues arise when reflectance values bottom out, such as during the calculation of NDVI or other indices involving the NIR band, which occurs when clouds temporarily during flight missions, exposing the terrain to direct sunlight. This issue of oversaturation in the NIR band was also reported by Wang^41^. While the DLS compensates for fluctuations in irradiance, it is effective only for global changes in lighting conditions. While the DLS compensates for fluctuations in irradiance, it is effective only for global changes in lighting conditions. The problem is exacerbated in dense forests, where obtaining shadow-free reference panels is nearly impossible, and capturing calibration data at different locations before and after missions is impractical. This could result in time differences from the actual flight mission, during which considerable changes in solar angle might occur.

The size of the reflectance panels also impacts the difficulty of radiometric calibration. Honkavaara et al.^49^ showed a better calibration for larger, custom-made reference panels of 1 × 1 m than the manufacturer’s provided method. Some studies have also demonstrated improved calibration methods using even larger reflectance tarps^50–52^. However, this does not alleviate the problem of acquiring calibration data in dense forests or the previously mentioned sudden changes in illumination. Therefore, further testing and development of improved field radiometric calibration strategies are imperative to more effectively utilize multispectral sensor capabilities.

Despite the challenges with multispectral sensors, particularly in the NIR band, the utility of the RGB bands is notable. Low-cost UAV setups with RGB sensors are widely available, facilitating the collection of vast data. This high data volume is crucial for developing models for various tree species in intensive monitoring plots. A key question is whether training data for models derived from visible bands need calibration from the multispectral sensor. In this case, the model trained with calibrated GCC generalized well with the uncalibrated GCC, but it remains to be seen if this holds true for new datasets and other tree species.

Errors can also arise from crown segmentation in pixel value extraction. For instance, branches from a neighboring tree with earlier phenological onset could overlap into the segmented crown area of the target tree. As segmentation is typically performed with a fully developed canopy (after phase 5.0), such overlapping branches are challenging to account for. Recording influential branches from neighboring trees during ground observations and excluding them from training datasets could improve the quality of training data.

The feature selection process in this research, especially partitioning training datasets by year for testing, was effective. It allowed for scrutinizing and removing training data portions that could affect model generalizability. For instance, the *br-ndvi-20-21* derived from multispectral sensors excludes the 2019 dataset due to its lower quality radiometric calibration, time differences between observations, a slightly different multispectral sensor, and a different observer for ground observations. Conversely, the *gcc-19-20* models generalized well with the 2019 datasets incorporated, using only bands from the visible spectrum. This suggests that the main factors in error propagation lie in the quality of radiometric calibration and sensor mixing with NIR bands, a conclusion that might not have been apparent without partitioning training by year. Interestingly, sensor mixing does not seem to be an issue with RGB imagery, which is advantageous for acquiring large data volumes.

Incorporating meteorological data, such as “warming” days (AIRTEMP), as a model feature suggests that other factors, such as a dynamic start date and chilling days, should also be considered for a successful phenological model in fusion with spectral data. However, this concept is somewhat limited, as meteorological data at the individual tree level might not explain the heterogeneity of individual trees in phenological development. The fusion of meteorological and spectral data is more suited for larger-scale applications, where phenological data are applied standwise rather than at the individual tree level.

Regarding the *Britzer* foliation method, translating ground observations into remote sensing data was not feasible. Consequently, the *Britzer* method of foliation has been abandoned at the Britz research station and replaced with the ICP Forests flushing method. Currently, the long-term *Britzer* phase method, alongside the flushing method, is conducted with the aim of simplifying observations and enabling harmonization of Britz research station data with the ICP Forests network at the international level.

## Conclusion and future outlook

This research focuses on a ML approach for predicting spring phenological phases of European beech using UAV multispectral data. Over three years (2019–2021), synchronous ground observations and UAV-derived multisensor indices were used to train and validate a variety of ML models. A comprehensive feature selection method was employed, incorporating Spearman correlation, polynomial fitting, and ML techniques.

The models were further evaluated using unseen data, and the effectiveness of various training data partitions by year was assessed to identify potential sources of error. The most effective combination of training data partition, vegetation index, and ML algorithm was found to be the 2019/2020 dataset, the Green Chromatic Coordinate (GCC), and GAM boosting. This model achieved a RMSE of 0.22 at the Kahlenberg site, 0.43 at the Black Forest site, and 0.54 with 2022 data at the Britz Research Station.

However, it was observed that the *Britzer* method of foliation could not be modeled successfully, with RMSE values significantly exceeding the 10% error threshold. The study's findings underscore the potential of a feature selection-based ML pipeline that leverages radiometrically calibrated visible bands. This approach is capable of predicting spring phenological phases using RGB imagery obtained from widely available, low-cost sensors. The research thus contributes to the advancement of accessible and accurate phenological modeling using UAV technology.

The *Britzer* phenological phase method is particularly valuable at intensive monitoring sites such as the Britz research station, but its complexity may not be as practical for use in external plots. In such cases, the ICP Forests flushing method presents a more suitable option. The integration of UAV-based data with the ICP Forests flushing method has the potential to enhance the existing datasets by providing predictions of phenology flushing on a stand-wise basis and over larger areas.

The developed concept could potentially be further developed to roughly predict later phases from the timing of phase 1. This would allow gap filling when assessments were missed. However, this is beyond the scope of this paper. But implementing large-scale mapping of phenological flushing could pave the way for training and validating models using data obtained from satellite platforms. This approach would enable the upscaling of phenological data, facilitating broad-scale mapping applications in forest phenology. Moreover, it would assist in creating historical phenological time-series maps, which are crucial for assessing the impacts of climate change from a spatial perspective.

Such advancements would not only improve the accuracy and scope of forest phenology monitoring but also contribute significantly to our understanding of how climate change affects forest ecosystems. This integration of various data sources and methods exemplifies the potential of combining traditional ground-based methods with modern UAV and satellite technologies for ecological research and monitoring.

Extensive research and experimentation remain necessary in this field, and several key areas are recommended for future investigation. First, there should be a focus on visible bands (RGB) and testing the feasibility of sensor mixing. This involves exploring the potential and limitations of combining different sensor data for more comprehensive analyses.

Another important aspect is the acquisition of training data, where efforts should be made to ensure an even distribution of phenological phases. Achieving this requires well-coordinated and synchronous cooperation with ground observation teams. In line with this, the development of models for additional tree species present in intensive monitoring plots is crucial. This would enhance the scope and applicability of the research.

Furthermore, adapting the ICP Forests flushing levels to UAV-based modeling on an international scale is essential. This adaptation process may involve regionalizing models to account for geographic and climatic variations. Special training data acquisition campaigns involving ground observation experts should be conducted to acquire more continuous flushing values (e.g., 5% levels) rather than relying solely on five code classes for model training. This approach would enable the use of regression-based ML models, which are often more effective than classification-based models and facilitate the collection of high-quality training data.

There is also a need to develop models that can predict other phenological phases or flushing levels from a single observation epoch. This capability is crucial for determining the accurate timing of when flushing or specific phases occur, moving beyond the limitations of statistical interpolation methods. Finally, translating observations or ML predictions to the stand level is significant for the development of models suitable for satellite applications. Such advancements would greatly benefit large-scale mapping and monitoring efforts, providing more precise and comprehensive insights into forest phenology.

## Data Availability

The datasets generated during and/or analysed during the current study are available from the corresponding author upon reasonable request.
